# The State of the World’s Beaches

**DOI:** 10.1038/s41598-018-24630-6

**Published:** 2018-04-27

**Authors:** Arjen Luijendijk, Gerben Hagenaars, Roshanka Ranasinghe, Fedor Baart, Gennadii Donchyts, Stefan Aarninkhof

**Affiliations:** 1 Faculty of Civil Engineering and Geosciences, Delft University of Technology, Delft, The Netherlands; 2 Deltares, Delft, The Netherlands; 3 IHE Delft, Institute for Water Education, Department of Water Science and Engineering, Delft, The Netherlands; 4 Water Engineering and Management, Faculty of Engineering Technology, University of Twente, Enschede, The Netherlands

## Abstract

Coastal zones constitute one of the most heavily populated and developed land zones in the world. Despite the utility and economic benefits that coasts provide, there is no reliable global-scale assessment of historical shoreline change trends. Here, via the use of freely available optical satellite images captured since 1984, in conjunction with sophisticated image interrogation and analysis methods, we present a global-scale assessment of the occurrence of sandy beaches and rates of shoreline change therein. Applying pixel-based supervised classification, we found that 31% of the world’s ice-free shoreline are sandy. The application of an automated shoreline detection method to the sandy shorelines thus identified resulted in a global dataset of shoreline change rates for the 33 year period 1984–2016. Analysis of the satellite derived shoreline data indicates that 24% of the world’s sandy beaches are eroding at rates exceeding 0.5 m/yr, while 28% are accreting and 48% are stable. The majority of the sandy shorelines in marine protected areas are eroding, raising cause for serious concern.

## Introduction

Coastal zones have historically attracted humans and human activities due to the abundant amenity, aesthetic value and diverse ecosystem services that they provide. As a result, the coastal zone all over the world has become heavily populated and developed^1–3^ with 15 of the 20 megacities (population >10 million) of the world being located in the coastal zone. The global coastline is spatially highly variable and comprises several different types of coastal landforms, some examples being barrier islands, sea cliffs, tidal flats, and river deltas. Of these different coastline types, here we focus on sandy coasts, which are highly dynamic in time and space, and constitute a substantial part of world’s coastline^4^. As sandy coasts are highly developed and densely populated due to the amenity and aesthetics that they provide, erosion of these coasts over the last few decades is already resulting in coastal squeeze^5^. Inevitably, climate change impacts on sandy coasts will only exacerbate this situation^6,7^. Thus, reliable assessments of the occurrence of sandy coasts and their rates of shoreline change are basic necessities for effective spatial planning, sustainable coastal development, coastal engineering projects, and mitigation of climate change impacts along high value coastlines around the world.

Despite the utility, economic benefits, and the dynamic nature of sandy coasts, there is no reliable global-scale assessment of their occurrence or rates of shoreline change (i.e. erosion/accretion rates) therein. Presently available global scale estimates of these phenomena vary widely, and the way in which most of these estimates have been derived is unclear at best. For instance, the percentage of occurrence for sandy shorelines worldwide reported in literature varies by a factor 7 ranging from 10%^8^ to 75%^9^. With regard to rates of change in sandy shorelines, several reliable recent regional scale estimates exist for Europe (27% eroding^10^ and the US East coast barrier beaches (86% eroding^11^. The only global scale assessment available is reported by Bird^12^ that estimated 70% of sandy shorelines worldwide were eroding. However, because Bird’s study, ground breaking as it was at the time, was primarily based on a survey of 200 participants from 127 countries, this estimate is rather qualitative.

Robust estimation of shoreline change rates by necessity requires continuous and long-term information on shoreline position. Historically, the acquisition of shoreline data sets has been a laborious and expensive task as it involved traditional land-based surveys or the analysis of temporally sparse data collected from aerial platforms (photographs or lidar). The increasing availability, resolution and spatial coverage of satellite imagery in recent years now provide a powerful alternative to derive reliable, global scale shoreline data as we demonstrate in this article.

The method commonly used to extract shorelines from satellite images in the past involved painstaking image by image analysis of series of overlapping images. The recent launching of the Google Earth Engine (GEE) platform, containing a continuously updated global satellite image archive, now enables efficient global scale shoreline detection. Having both a petabyte satellite image collection and parallel computation facilities combined on the server side of the platform reduces image processing time to only several minutes per image^13^ and enables efficient validation of the automatically detected shorelines at multiple sites where ground-truth field data are available.

To enable global mapping of sandy shorelines it is first necessary to identify sandy beaches and then determine shoreline positions in every image in the GEE platform. The spatio-temporal scales associate with this study (i.e. global scale, 33 year analysis) and the large amount of satellite images that therefore need to be analysed necessitates the use of robust automated image analysis techniques. Machine learning^14^ and image processing^15^ techniques that lend themselves to such automated analyses are readily available. However, to be able to use satellite derived shoreline positions for real-world applications such as reliably estimating trends and structural damage to infrastructure, a horizontal resolution of at least 10–20 m is required. For example, shoreline change rates above 0.5 m/yr over a long period are typically employed to flag a coastal area as one experiencing chronic (=long term i.e. decades to centuries^7^) erosion or accretion. Over a period of 30 years that would mean a total displacement of just 15 m. Previous studies have evaluated the positional accuracy of satellite derived shorelines (SDS) based on single images^15–19^ to range between 1.6 and 10 m. It should be noted that these studies suffered from limitations such as the number of images used, the quality of the *in-situ* data used for validation or the magnitude of changes in observed shoreline position. Recently, Hagenaars *et al*.^20^ presented a long-term, but local-scale satellite image analysis on shoreline trends, that overcomes all of the aforementioned limitations. They found the accuracy of the SDS derived from moving average composite images to be of subpixel precision (~half a pixel size, i.e., 15 m for Landsat and 5 m for Sentinel-2). The accuracy of <15 m, reported by Hagenaars *et al*.^20^ for composite Landsat images, matches the required displacement of 15 m for reliable shoreline change classifications over the last 30 years. For that reason, we adopt the same approach in this study, yet at a global scale.

Here we present an up-to-date global-scale assessment of dynamics of sandy shorelines using a fully automated analysis of 33 years (1984–2016) of satellite images. First, we detect sandy beaches worldwide by applying a pixel-based supervised classification to a cloud-free high-resolution global composite image for 2016. A digital beach training dataset is provided to the classification software and validated for 50 locations worldwide that include both sandy and non-sandy beaches. Next, we apply a shoreline detection algorithm to cloud free global annual composite images using more than 1.9 million historical Landsat images. After a successful quantitative validation of this technique at multiple sites located in various geographical settings and environmental conditions, we derive shoreline change rates in m/yr at transects with an alongshore spacing of 500 m along the world’s shoreline. The above mentioned methods are elaborated in the Methods section below while the complete validation is presented Supplementary Material (S2).

The main outcomes of our analysis include: (a) the global occurrence of sandy beaches, (b) rate of erosion/accretion at all sandy beaches in the world, (c) highlights of observed natural and human induced impacts on coastal erosion/accretion at selected locations, and (d) identification of global hot spots of coastal erosion/accretion.

## Results

### Global Occurrence of Sandy Shorelines

Coastal classifications have been widely employed in the field of geomorphology to characterise the diversity of coastal landforms and the contexts within which they emerge, but hitherto no single system of classification has been comprehensive in scope or coverage^21,22^. Criteria in these classifications typically include tectonic^23^ and hydrodynamic controls, as well as the sedimentological response. Hydrodynamics controls considered include classifications of wave parameters^24^, tidal range^24,25^ and a combination of both^26^. A ternary classification presented by Boyd *et al*.^27^, which considers the relative importance of fluvial inputs, wave energy, and tidal forcing provided a useful analysis of siliciclastic sedimentary coasts. The combination of tectonic and hydrodynamic controls led to the proposition of coastal morphogenetic classifications^28^, which are probably the most widely used classification schemes.

Sediment texture and composition^29^ are additionally useful to classify and describe coastal sedimentary environments. However, previously reported values of the global occurrence of sandy shorelines vary between 10% and 75% (see Table 1). The methods used to arrive at these values remain, in most cases, unclear or qualitative (as also indicated in Table 1).

**Table 1:** Reported values of global and regional occurrences of sandy shorelines and percentages of chronic erosion and accretion.

Region	Parameter	References	Method used	Reported values		Derived values
Global	Percentage of sandy shoreline	Bird^12^	Interviews	20%		31%
		Bird^4^	Not stated	30%		
		Inman & Nordstorm^23^	Not stated	11%		
		Hardisty^54^	Not stated	34%		
		Van Rijn^8^	Not stated	10–15%		
		Bascom^9^	Not stated	75%		
		Brown^55^	Not stated	67%		
		Durgappa^56^	Not stated	20%		
		Bird^38^	Not stated	40%		
		Hinkel *et al*.^3^	Not stated	11%		
	Percentage of eroding sandy shoreline	Bird^12^	Interviews	Accretion	10%	27%
			Interviews	Stable	20%	49%
			Interviews	Erosion (<−0.5 m/yr)	70%	24%
			n.a.	Intense erosion		16%
			n.a.	Severe erosion		7%
			n.a.	Extreme erosion		4%
Europe	Percentage of sandy shoreline (sandy shoreline length)	Eurosion^10^	Aerial photos & surveys	40% (40,000 km)		23% (31,000 km)
	Percentage of eroding sandy shoreline	Eurosion^10^	Aerial photos & surveys	27% (excluding uplift of Finland and Sweden)		28%
USA	Percentage of sandy shoreline	Short^43^	Not stated	33%		30%
	Percentage of eroding sandy shoreline (Atlantic and Gulf coast only)	Heinz Center^44^	Aerial photos	80–90%		52%
Australia	Percentage of sandy shoreline	Woodroffe *et al*.^57^ Short incl. Tasmania	Not stated	43–49%		52%
	Percentage of eroding sandy shoreline	No source found	n.a.	Not reported		25%

In our analysis, we applied supervised (human-guided) classification to global cloud-free satellite images (see Section 3.2) to identify sandy shorelines. One of the main reasons for our focus here on sandy beaches is that detecting shoreline dynamics for non-sandy shores like muddy coasts can be complex. Mild foreshore slopes, resulting in large horizontal tidal excursions, and high water content hampers correct shoreline detection. In the case of mangroves, seasonal growth cycles can impede correct shoreline detection. Moreover, it should be noted that as the reflectance signatures of sand and gravel beaches cannot be differentiated in the satellite imagery, as both materials originate from the same granular composites of finely divided rock, our references to sandy beaches herein also includes gravel beaches.

**Figure 1:**
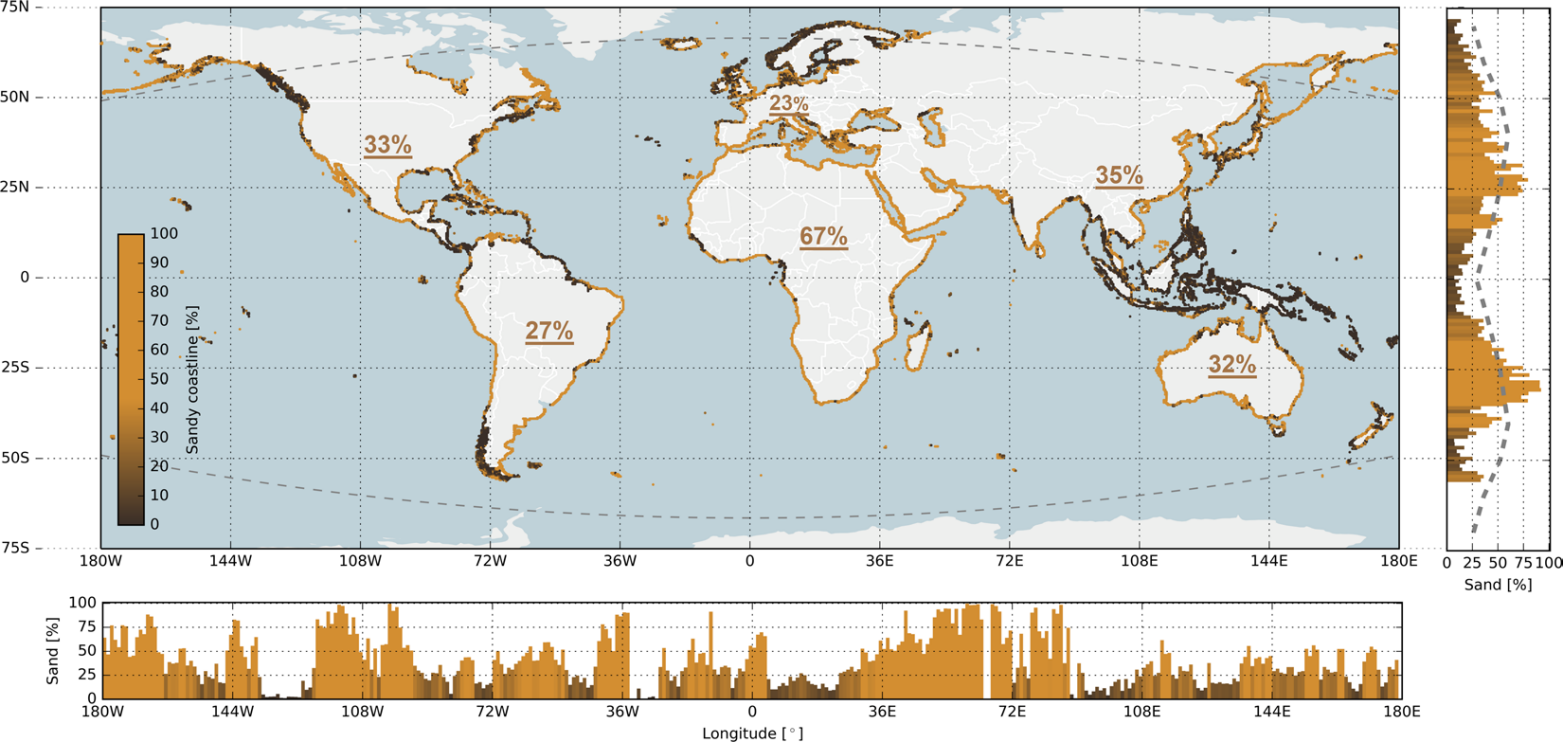
Global distribution of sandy shorelines; the coloured dots along the world’s shoreline represent the local percentage of sandy shorelines (yellow is sand, dark brown is non-sand). The subplot to the right presents the relative occurrence of sandy shorelines per degree latitude, where the dashed line shows the latitudinal distribution of sandy shorelines reported by Hayes^31^. The lower subplot presents the relative occurrence of sandy shorelines per degree longitude. The curved, dashed grey lines in the main plot represent the boundaries of the ice-free shorelines considered in our analysis. The underlined percentages indicate the percentages of sandy shorelines averaged per continent. Map is created with Python 2.7.12 (https://www.python.org) using Cartopy (v0.15.1. Met Office UK. https://pypi.python.org/pypi/Cartopy/0.15.1) and Matplotlib^58^.

Our analysis showed that 31% of the ice-free world shoreline is sandy. The continent with the highest presence of sandy beaches is Africa (66%), while in Europe only 22% of the shoreline is sandy (see inserted table in Fig. 1). The percentage of sandy shorelines obtained from this analysis for USA and Australia compare well with the more recently reported regional scale values (see Table 1). The larger deviation in percentage found for Europe is significantly influenced by the smaller total length of shoreline used in the Eurosion^10^ data base. It should be noted that the sandy beach classification also includes the gravel beaches in the world. The reflectance signatures of sand and gravel beaches cannot be differentiated in the satellite imagery as both materials originate from the same granular composites of finely divided rock.

The global latitudinal distribution of sandy shorelines shows a distinct relation with latitude and hence with climate; no relation is found with longitude. The relative occurrence of sandy shorelines increases in the subtropics and lower mid-latitudes (20°–40°) with maxima around the horse latitudes (near 30°S and 25°N; see Fig. 1). In contrast, they are relatively less common (<20%) in the humid tropics where mud and mangroves^30^ are most abundant as a result of high temperatures and rainfall. The percentage of sandy shorelines decreases beyond the 50° parallel. This latitudinal distribution of sandy shorelines is in line with the latitudinal variation of the common sediments in the inner continental shelf reported by Hayes^31^, based on ~2000 transects from 131 coastal areas (see right subplot in Fig. 1).

### Global sandy beach erosion

Worldwide beach erosion became apparent during the 1980s following the studies of the International Geographical Union working group on the Dynamics of Coastal Erosion (1972–1976) and the Commission on the Coastal Environment (1976–1984). In these studies, two hundred participants representing 127 countries contributed to a survey which indicated that 70% (10%) of the world’s sandy beaches experienced net erosion (accretion) while 20% were stable^32^. However, as these estimates were primarily a result of interviews, they are necessarily qualitative, at best. Furthermore, the estimates likely did not take into account changes occurring along undeveloped and uninhabited coasts due to the subjective methodology adopted.

The quantitative global distribution of sandy shorelines presented herein, for the first time, allows the derivation of objective and up to date global scale assessment of chronic shoreline changes (i.e. beach erosion/accretion). Beach erosion can occur at a range of timescales^33^. Individual storms will generally result in rapid short-term erosion, followed by short-term accretion, leading to negligible net change over time scales of a few weeks-months. If sediment deficiencies persist for long periods of time (e.g. due to longshore gradients in sediment transport, reduction of fluvial sediment supply to the coast), chronic erosion can result. The analysis presented here focusses on such chronic erosion and accretion. However, there are no common standards for the classification of rates of chronic beach change^34^ which is generally quantified through some statistical treatment of erosion rates and/or volumetric losses (e.g. ref.^35^.

The accuracy of the SDS data of ~0.5 pixel (see Section 1) and the study period of ~30 years allows for a classification of beach change rates with class boundaries of 0.5 m/yr. Hence, we adopted the chronic beach erosion classification scheme proposed by Esteves and Finkl^36^ and extended it with a classification for extreme erosion resulting in the below scheme:

- Accretion>0.5 m/yr
- Stable−0.5 to 0.5 m/yr
- Erosion−1 to −0.5 m/yr
- Intense erosion−3 to −1 m/yr
- Severe erosion−5 to −3 m/yr
- Extreme erosion<−5 m/yr

Our assessment shows that 24% of the world’s sandy beaches are persistently eroding at a rate exceeding 0.5 m/yr over the study period (1984–2016), while 27% are accreting (see Table 1). About 16% (18%) of sandy beaches are experiencing erosion (accretion) rates exceeding 1 m/yr.

**Figure 2:**
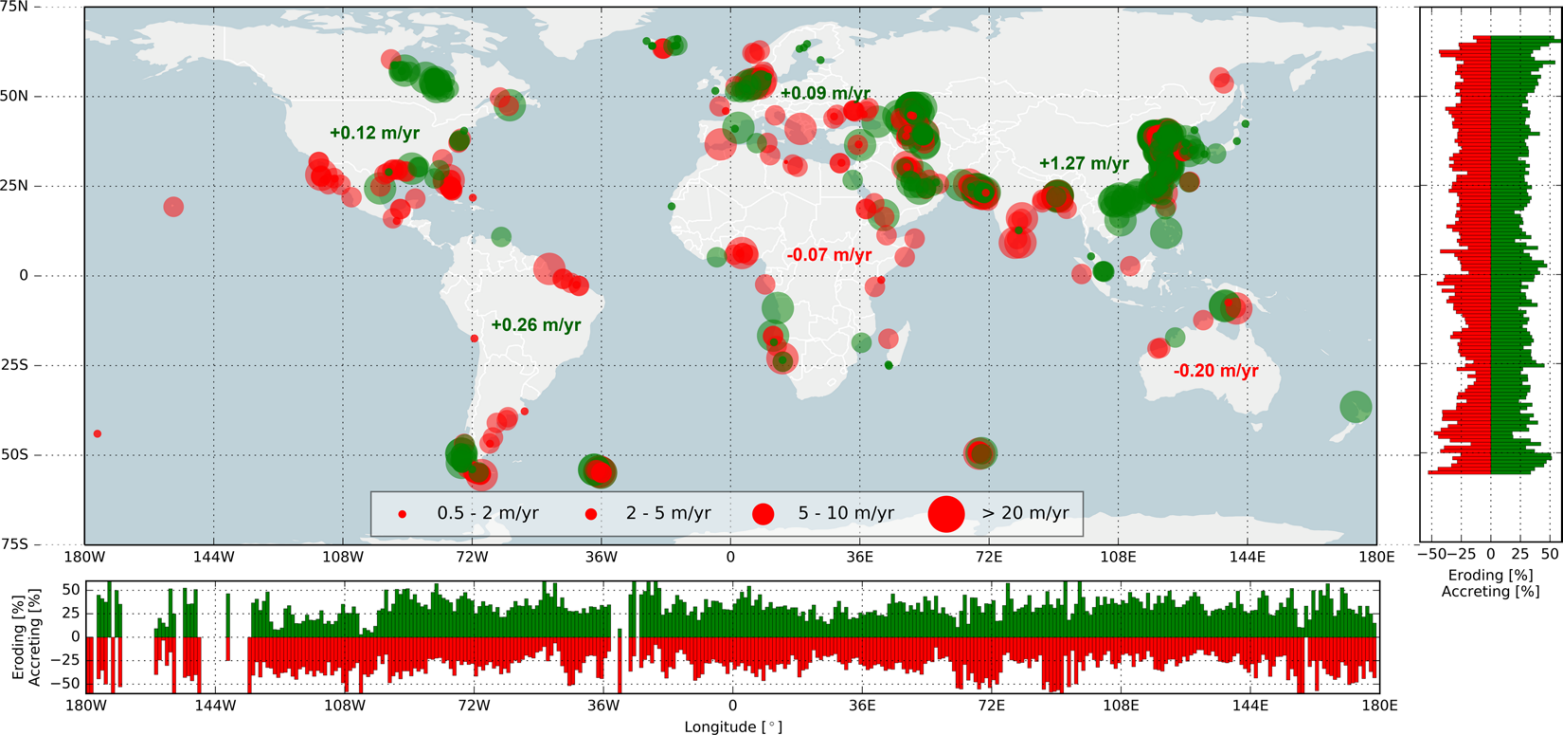
Global hotspots of beach erosion and accretion; the red (green) circles indicate erosion (accretion) for the four relevant shoreline dynamic classifications (see legend). The bar plots to the right and at the bottom present the relative occurrence of eroding (accreting) sandy shorelines per degree latitude and longitude, respectively. The numbers presented in the main plot represent the average change rate for all sandy shorelines per continent. Map is created with Python 2.7.12 (https://www.python.org) using Cartopy (v0.15.1. Met Office UK. https://pypi.python.org/pypi/Cartopy/0.15.1) and Matplotlib^58^.

Chronic erosion of beaches (<−0.5 m/yr) is shown across the globe with relatively low latitudinal variation (see Fig. 2). Generally, between 30% and 40% of sandy beaches per degree latitude are eroding with relatively high eroding values up to 50% just south of the equator associated with large-scale land losses adjacent to the Amazon River mouth.

More severe erosion rates are found at various locations across the globe. About 7% of the world’s sandy beaches experience erosion rates classified as severe. Erosion rates exceed 5 m/yr along 4% of the sandy shoreline and are greater than 10 m/yr for 2% of the global sandy shoreline. On the other hand, about 8% of the world’s sandy beaches experience significant accretion (>3 m/yr), while 6% (3%) are accreting more than 5 m/yr (10 m/yr).

Taking a continental perspective, Australia and Africa are the only continents for which net erosion (−0.20 m/yr and −0.07 m/yr respectively) is found, with all other continents showing net accretion. The continent with the largest accretion rate (1.27 m/yr; see table in Fig. 2) is Asia, likely due to the artificial development of the Chinese coast and large land reclamations in, for example, Singapore, Hong Kong, Bahrain and UAE. On a global scale, the world’s beaches have accreted on average 0.33 m/yr over the past three decades, i.e. a total gain of 3,663 km^2^ over this period.

Using the SDS data we then focussed on coastlines that are internationally recognised as nature protected areas by the World Database on Protected Areas (WDPA), which is the most comprehensive global database on terrestrial and marine protected areas, produced by UNEP-WCMC and IUCN^37^. Compared to the global average, a relatively high percentage of sandy shorelines in the WDPA-identified areas are experiencing erosion. Our analysis indicates that 32% of all marine protected shorelines are sandy of which 37% are eroding at a rate larger than 0.5 m/yr, while 32% are accreting.

### Quantifying local scale erosion/accretion due to human interventions

No single explanation can easily account for the observed erosion/accretion trends along the global sandy shoreline, or for the acceleration of erosion/accretion on any particular beach^38^. However, analysis of local trends derived from the global scale shoreline assessment presented herein can help identify natural and human drivers of shoreline change. To illustrate this, we present two highlights of erosive behaviour and two of accretive behaviour. Another four highlights are presented in the Supplementary Material (S3).

#### a) Sand mining and subsidence

**Figure 3:**
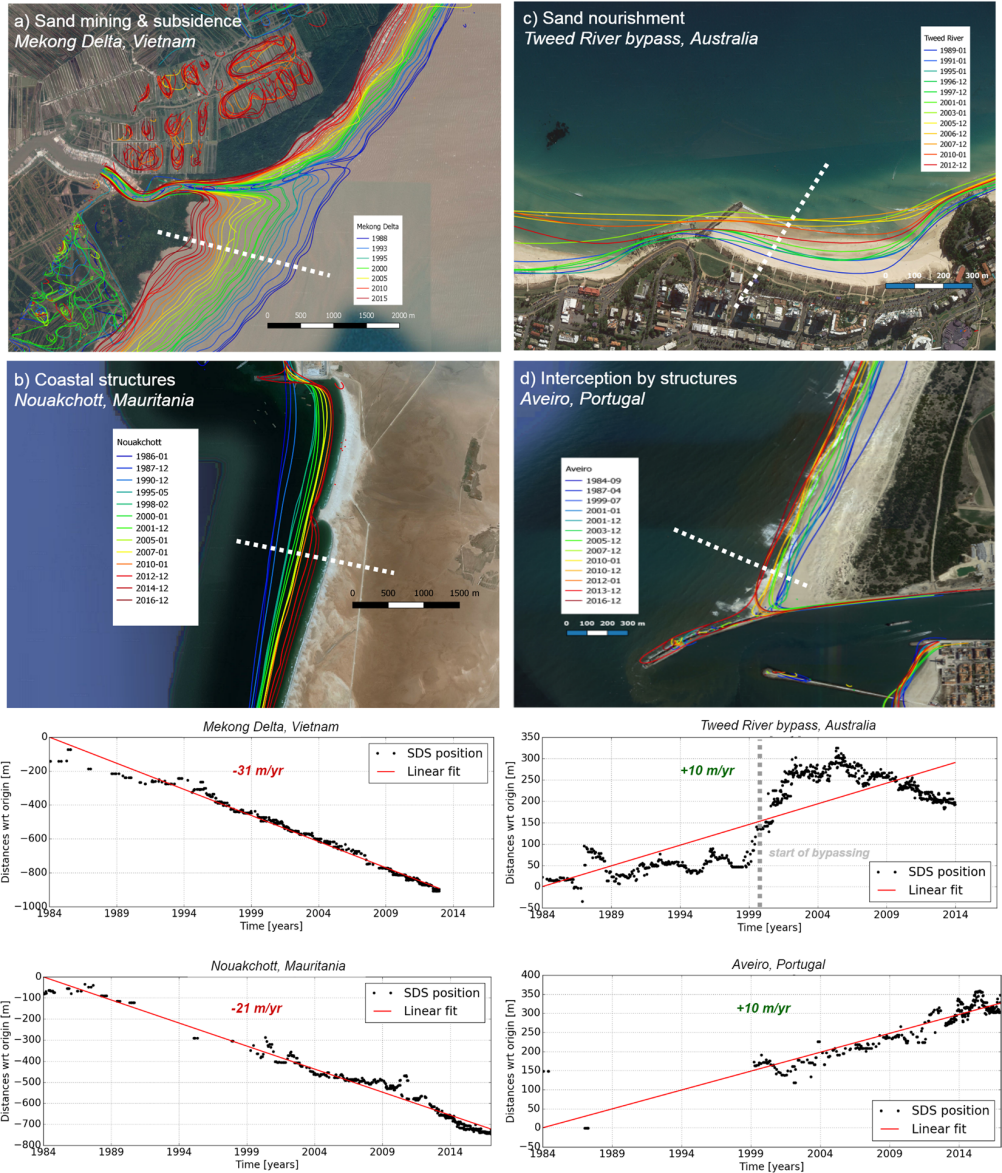
Examples of the satellite derived shorelines for four selected cases of beach erosion and accretion due to human interventions. The left column presents two erosive cases while the right column shows two accretive cases. In each figure, the blue line indicates the oldest SDS shoreline while the red line is the most recent SDS shoreline. The graphs below indicate the shoreline positions over time at the white dashed transect for each case; the upper graphs correspond to the images on the upper row. The indicated change rates (m/yr) are obtained from fitting a line-of-best fit to the shoreline position data for each transect. Figure is created with Python 2.7.12 (https://www.python.org) using Matplotlib^58^. Maps are created with QGIS version 2.18.3 (Open Source Geospatial Foundation Project, http://qgis.osgeo.org) using satellite images provided by Google Maps. Map data: Google, Terrametrics, CNES/Airbus, IGP/DGRF, and DigitalGlobe.

The Mekong Delta in Vietnam, the third largest delta in the world, is increasingly affected by human activities and exposed to subsidence and coastal erosion. The large-scale shoreline erosion is attributed to excessive sand mining in the river and delta channels, and subsidence due to unregulated groundwater extraction^39^. Analysis of the SDS data (Fig. 3a) reveals slight erosion between 1984 and 1990, after which higher, but steady erosion rates are found. Erosion rates in the considered area typically range between 25–30 m/yr over the last three decades. Based on the strong linear trend, the SDS data may be used for projections of land loss and displacement strategies, as it is not expected that erosion rates will decrease in the near future unless mitigating measures are implemented.

#### b) Coastal structures

The harbour structures at Nouakchott, Mauritania, blocked the large unidirectional north-south longshore transport of sand since 1986, causing areas of beach erosion that has impacted the local social and urban developments. The shoreline evolution rates observed after the harbour construction are 10 times larger than the values that would have been observed in the natural state^40^. The harbour breakwaters induced severe erosion over a distance of more than 10 km in the downdrift zone where accretion was likely to occur in the absence of the harbour. The SDS data (Fig. 3b) shows erosion rates of 20 m/yr.

#### c) Sand Nourishments

A large-scale bypass system became operational in 2001 at the Tweed River, New South Wales, Australia, to mitigate erosion of the beaches to the north of jetties constructed at the river entrance^41^. The bypass system pumps sand from south of the river mouth to three beach compartments located north of the river through buried pipelines. The SDS data (Fig. 3c) depicts a beach widening of ~250 m at Coolangatta Bay in the four years after the bypass system was commissioned.

#### d) Interception of longshore drift by coastal structures

The construction of two training breakwaters at Praia da Barra near the Aveiro Lagoon, Portugal interrupted the high southward ambient alongshore transport estimated at about 1 million m^3^/yr^42^. This resulted in erosion at the south of the trained inlet affecting the shoreline over about 30 km downdrift, but also strong accretion updrift. The SDS data reveals the continuous and ongoing accretion of the northern beach at a rate of about 10 m/yr (Fig. 3d).

### Global hot spots of erosive and accretive beaches

Here we present the top eroding and accreting coastal stretches (i.e. hot spots) in the world (Table 2). The largest erosive hot spot is just south of Freeport in Texas where a 17 km stretch the beach has eroded on average more than 15 m/yr over the last three decades. The world’s longest coastal stretch suffering severe erosion is located farther to the east in Texas where we observed a 29 km stretch of sandy beach with a mean erosion rate of 5.3 m/yr. Interestingly, four of the seven largest hot spots are located in the USA, consistent with the widespread concern and reports of erosion in the USA^11,35,43,44^.

**Table 2:** World’s largest erosive and accretive sandy beach hot spots.

	Areal change Rate (m^2^/yr)	Mean change rate (m/yr)	Length of section (km)
**Erosive Hot Spot Beaches**			
Freeport, Texas, USA	−258,678	−15.2	17
San Rafael National Park, Chile	−243,459	−8.4	29
Rockefeller reserve, Louisiana, USA	−192,758	−16.0	12
Nebel island, Germany	−175,716	−12.1	15
Esbjerg, Denmark	−162,695	−8.1	20
High Island, Texas, USA	−155,287	−5.3	29
Hog Island, Virginia, USA	−154,848	−13.5	12
**Accretive Hot Spot Beaches**			
Diamond mines, Oranjemund, Namibia*	219,748	8.8	25
Around Karachi Port, Pakistan*	203,752	13.1	16
Schiermonnikoog Island, Netherlands	194,752	9.7	20
Rijnland Coast, Netherlands*	190,105	12.3	16
South Coast of Madagascar	153,573	7.0	22
Port Said, Egypt*	149,133	13.0	12
Mauritania	140,239	6.9	22

Areal change rate is calculated by multiplying the length of the section with the mean of the shoreline change trends of all transects in the relevant coastal stretch. The human-induced accretive hot spots are indicated by an asterisk.

The largest accretive hot spot is in Namibia at a location where a mining company has built unprotected sandy bunds in the sea to facilitate the diamond prospecting. The area landward of the bunds is dried out to enable more convenient diamond prospecting. Naturally accreting beaches of lengths exceeding 20 km and change rates larger than 7 m/yr are found at a migrating barrier island (Schiermonnikoog, The Netherlands) and at locations where sand dunes migrate into the sea (Madagascar and Mauritania). It is noteworthy that four of the seven largest accretive hot spots are in fact human-induced.

### Outlook

In the near future we foresee great potential for remote sensing techniques and big data analysis in operational monitoring of the World’s coast and beaches. The global sandy shoreline change analysis presented herein is primarily based on Landsat imagery with 30 m resolution and a revisit time of 16 days. In recent years new satellites (Sentinel-2a,b) that will significantly enrich the satellite imagery data both in temporal (revisit time of a few days) and spatial resolution (<10 m) have been launched. At present, private institutions already provide satellite images at approx. 1 m resolution with a daily revisit and global coverage. We expect that this trend will demand more emphasis on big data statistics in the near future to closely and better monitor how the planet is changing.

## Methods

**Figure 4:**
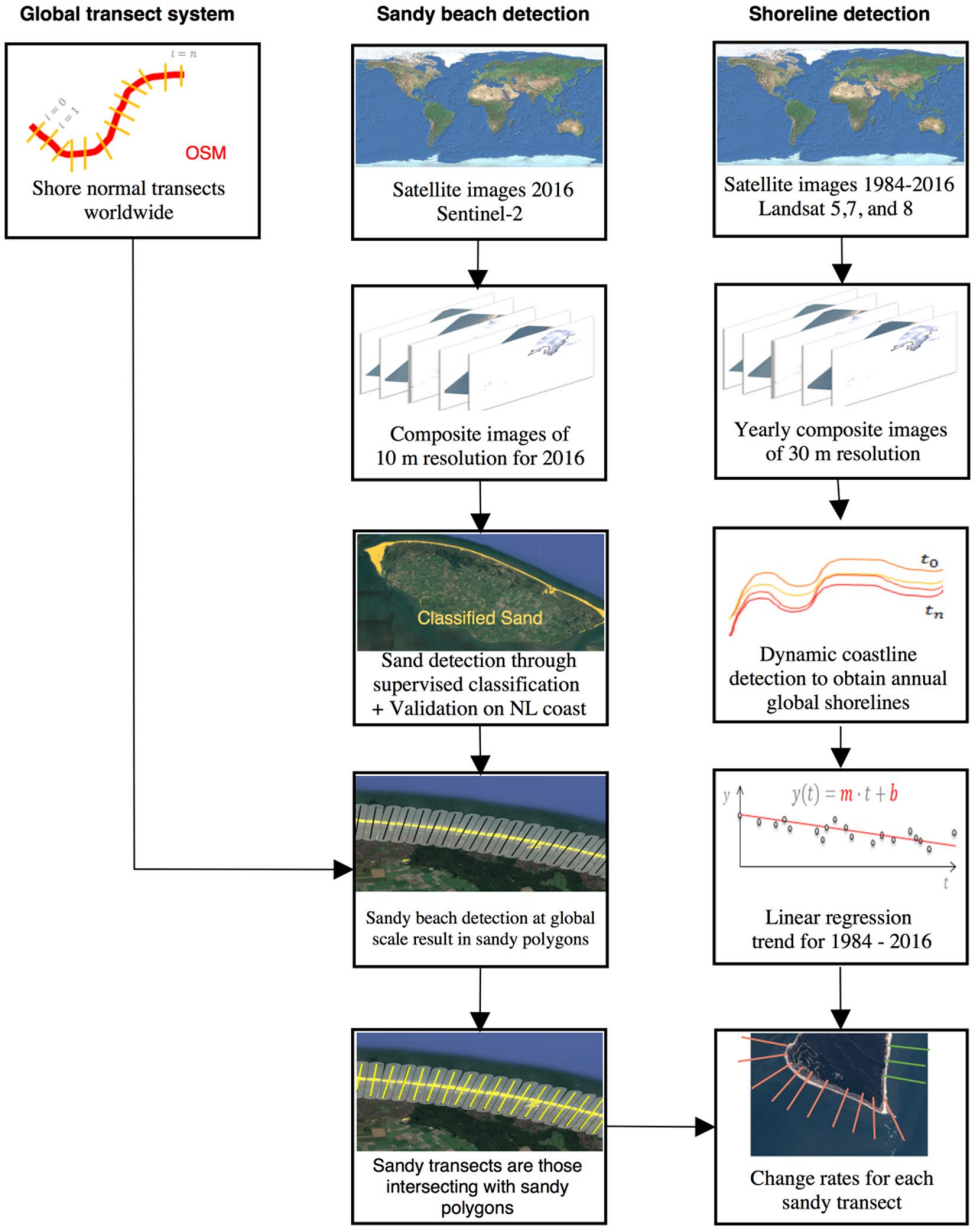
The procedure followed for deriving shoreline change trends for sandy shorelines using a global transect system. The figure is compiled using www.draw.io, while the maps in the figure are made using © Mapbox and © OpenStreetMap, available under the Open Database License (https://www.openstreetmap.org/copyright).

The workflow applied in this study comprises three methods as discussed below and illustrated in Fig. 4.

### Global transect system

For global analysis and visualization purposes, we defined 500 m spaced transects orthogonal to the global shoreline from the OpenStreetMap^45^ (OSM) dataset of 2016. The length of the global shoreline, as well as per country, are calculated by summing straight intercepts between the transects. The total length of the world’s ice-free shoreline determined from this analysis is 1.11 million km, which is comparable with previously reported values of 1 million km^3^, 1.16 million km^46^, and 1.47 million km^47^. In the future, we intend to merge the 500 m transect system with locally available grids and refine it where appropriate.

### Detection of sandy beaches

Sandy beaches are detected by applying a pixel-based supervised classification to a global Top of Atmosphere (TOA) reflectance percentile composite image for the year 2016 using all available Sentinel-2 images. To facilitate this, the world has been divided into boxes of 20 km × 20 km. Using the 2016 OSM shoreline, we only select the boxes that intersect with the 2016 shoreline, which results in about 24,000 boxes to be analysed. To train the supervised classifier, a beach area consisting entirely of sand is selected (at the Dutch Texel island) as well as training areas on land representing different types of land use. To select the most promising classification algorithm, the validation results were quantitatively compared to the sandy *beach feature* in OSM. From the four considered classification algorithms, the Classification and Regression Tree (CART) classifier resulted in the lowest omission error and the highest percentage of true positives (97%) using the beach features in a 100 km long section of sandy beaches along the Dutch coast.

Next, we apply the trained supervised classification method to all boxes to detect sandy beaches at global scale as the OSM *beach feature* is not available for the entire globe. A search area of 500 m land- and seaward of the 2016 OSM shoreline is defined, after which the supervised classification is conducted using GEE to automatically detect sandy beaches. The result is a series of polygons encapsulating all sandy beaches worldwide, including both quartz and carbonate sands, and gravel. More than 50 sand validation locations, randomly spread across the world, were selected independently from the training dataset. Validation through visual inspection resulted in 96% accuracy (see Supplementary Material S1).

Transects that intersect with a sandy polygon are classified as ‘sand’ and others as ‘non-sand’. Transects for which no sand classification could be made due to the absence of a cloud-free Sentinel-2 image are labelled as ‘undetermined sediment composition’. As this is applicable for 5.2% of all transects the percentage of sandy beaches is 31% ± 1.5%, assuming that the unknown areas behave similar to the global mean.

### Dynamic shoreline detection

To remove the effects of clouds, shadows, snow, and ice, we generate yearly top-of-the-atmosphere reflectance composites, which we then use to estimate an accurate surface water mask using dynamic thresholding method described in ref.^48^. Yearly composite images generated by the 15% reflectance percentiles per pixel were analysed to determine global shoreline positions, resulting in the removal of clouds and shadows. This approach is comparable to how Hansen^49^ generates composite images. However, the use of an exact percentile value turns out to be more suitable than the interval mean averages used in that study. Analysis of the composite images significantly decreases the influence of the tidal stage on the detected shoreline positions and averages out seasonal variability in wave and beach characteristics. Nevertheless, at sites with persistent swell conditions the wave-induced foam due to wave breaking will introduce a seaward offset in detected shorelines. Fortuitously, however, this persistency ensures that the wave-induced offset is most likely also present in annual composites and shorelines of other years. Thus, the wave effects on detected shorelines are likely to be limited, especially where long-term shoreline change rates at such sites are concerned.

For validation purposes with long-term *in-situ* shoreline changes, an optimal averaging period of 192 days is applied; i.e. the first integer that is found when dividing the global revisiting time of the satellite sensor (16 days) by a semidiurnal tidal period (approx. 12 hrs). In case all satellite images in this averaging interval are cloud-free the average water level corresponds to mean sea level. The potential year-to-year random deviation from ‘mean sea level’ due to omitted satellite images is assumed to have a limited effect on the 33-year trend of shoreline change; this assumption will be verified as part of further research.

Next, the resulting composite images are used to estimate the Normalized Difference Water Index (NDWI). The Canny edge detection filter is used to roughly estimate the position of the water-land transition, followed by the use of the Otsu thresholding method^50^ on a buffer polygon around the water-land transition to identify the most probable threshold to classify water and land on the image. The detected water lines at the edge of the water mask are smoothed using a 1D Gaussian smoothing operation to obtain a gradual shoreline avoiding the pixel-induced staircase effect. A value of three gives the best results based on the four validation cases; meaning that it takes three cells on both sides during the 1D smoothing. The method may result in several shoreline vectors since lakes and small channels are detected. In this case, only the most seaward shoreline position is analysed.

Other studies have applied global surface water change and occurrence detection^48,51^ but they lack validation with *in-situ* measured shoreline changes. A number of studies have validated their methods with either cross-shore positions at one location^19^ or over limited spatial scales^15^. Here we evaluate the validity of the shoreline detection method for four cases representing different types of beaches, sand, tidal and wave characteristics. Given the geographical spreading, we selected the following beaches with long-term shoreline monitoring programs: the Sand Engine (The Netherlands), Long Beach, WA (West Coast, USA), Narrabeen (Australia) and Hatteras Island (East Coast, USA). The latter case is presented below while the others are presented in the Supplementary Material (S2).

### Hatteras Island validation

Validation of the shoreline detection method with observed shoreline changes was conducted along 63 km of sandy shoreline of Hatteras Island, North Carolina, spanning 13 years^52^. The measured shorelines used in the analysis were generated from georeferenced historical aerial photographs and are used to develop shoreline change rate indicators for Hatteras Island, from Oregon Inlet in the North to Cape Hatteras in the South. A total of nine aerial photographs, covering the period ranging from 1989 through 2002, were obtained by the U.S. Army Corps of Engineers Field Research Facility in Duck, North Carolina. The high water line shorelines were digitized to produce a time series of shorelines for the study area. Rates of shoreline change were calculated for 1989–2002 using linear regression.

**Figure 5:**
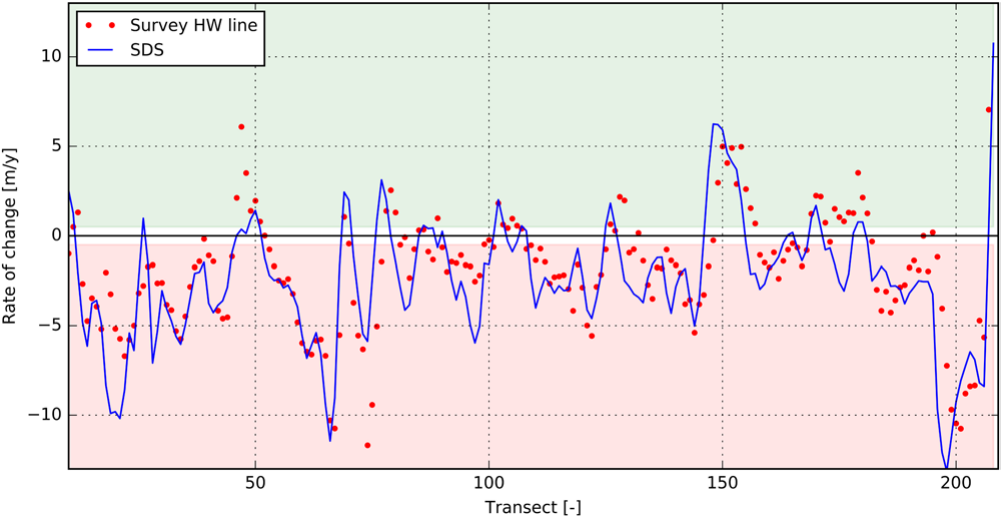
Observed trend rates (red dots) and satellite-derived trend rates of shoreline change (blue line) along Hatteras Island for the period 1989–2002. Figure is created with Python 2.7.12 (https://www.python.org).

For the same time period we collated 325 cloud-free satellite images and determined the shoreline position for this coastal stretch; the analysis took only 8 hours in total due to the computational power of the GEE platform. For each transect a linear regression was performed. The linear trends calculated from the SDS show good agreement with the observed shoreline change rates (see Fig. 5). The mean offset for all transects between observations and SDS is 2.0 m with a RMSE of 17 m.

The Supplementary Material (S2) summarizes the error statistics for all four cases. Based on these validations, the shoreline detection method can be concluded to be capable in deriving long-term shoreline change rates for a variety of coastal settings. The average of the offsets over three validation sites is 2.3 m with a RMSE of 21 m.

Although the quantitative evaluation of the applied shoreline detection method with *in-situ* observations shows good capabilities, more verification is essential. Unfortunately, however, quantification of the influence of macro-tidal ranges, wave breaking and run up, beach slopes, etc. requires tidal, wave and beach characteristic information, which are generally not freely available.

### Global change rates for sandy shorelines

For the global application presented here, we generated cloud-free annual-composites using the historical Landsat image archive. The automated shoreline detection method produces 33 annual global shorelines (1984–2016) with an alongshore resolution of 30 m. We then specified transects at a 500 m alongshore spacing, and determine the intersection point of each transect with the aforementioned annual shorelines, which provides a sequence of shoreline positions per transect. The shoreline change rate (m/yr) at each transect is then computed by applying linear regression to all shoreline positions at that location. Ideally, a SDS position is available for each transect annually. However, the availability of satellite images and cloud cover can limit the number of SDS positions. Encouragingly, however, 82% of all sandy transects consist of more than ten annual shoreline positions between 1984 and 2016. Nevertheless, to avoid unrealistic shoreline change rates we applied the following filters to all sandy transects:

- Transects containing less than 5 (out of 33) SDS data points as well as transects with a temporal coverage shorter than 7 years are omitted from the analysis (9% of all transects).
- Transects located beyond latitudes 60°N and 50°S (including Greenland and Antarctica) are omitted from the analysis due to possible ice coverage (9% of all transects).
- In the linear regression, outliers are identified as SDS points deviating more than three times the standard deviation and hence not considered in the regression. If the remaining number of data points is smaller than 5 points, then the transect is omitted from the analysis.

Applying these filters reduce the global data set to 81% of the original number of sandy transects. The linear regression method used to quantify long-term shoreline change rates performs well in capturing trends of chronic sandy shoreline change which is in line with findings of Crowell *et al*.^53^. However, multiple transects were characterised by unsteady changes in SDS positions for which other methods may be more appropriate. Ultimately, more than 60% of the 2.2 million transects show an uncertainty bandwidth of less than 50% of the linear trend rate, which can be considered as a proxy for the representativeness of the linear regression method.

The shoreline change rates, presented at an alongshore resolution of 500 m along the world’s shoreline, will become publicly available and be accessible through the interactive website at: http://shorelinemonitor.deltares.nl.

### Defining Hot Spots

In order to avoid localized hot spots, it was ensured that each eroding/accreting hot spot comprised at least 5 km of sandy shoreline where all considered transects showed either erosive or accretive change rates larger than 0.5 m/yr over the 33 year data set.

Two large-scale land reclamations appear in the top seven accretive beaches in the world. One reason is that those land reclamations consisted of bare sand in 2016, and hence are recognised as a wide sandy beach area by our methodology. The other reason is that the adjacent shorelines have advanced either due to the beach nourishment schemes or natural accumulation of sand in the shadow zones of these interventions.

## Electronic supplementary material

Supplementary material

## References

[1] Small, C. & Nichols, R.J. A Global Analysis of Human Settlement in Coastal Zones. J. of Coastal Research (2003).

[2] Hallegate S, Green C, Nicholls RJ, Corfee-Morlot J (2013). Future flood losses in major coastal cities. Nature Climate Change.

[3] Hinkel, J. *et al*. A global analysis of erosion of sandy beaches and sea-level rise: An application of DIVA. Global and planetary change. Vol. 111 (2013).

[4] Bird, E. C. F. Beach management. Chichester: John Wiley & Sons (1996).

[5] Pontee, N. Defining coastal squeeze: A discussion. Ocean & Coastal Management, Vol 84 (2013).

[6] Nicholls, R. J. *et al*. Coastal systems and low-lying areas. Climate Change 2007: Impacts, Adaptation and Vulnerability. Contribution of Working Group II to the Fourth Assessment Report of the Intergovernmental Panel on Climate Change, M. L. Parry, O. F. Canziani, J. P. Palutikof, P. J. van der Linden & C. E. Hanson, Eds, Cambridge University Press, Cambridge, UK, 315–356 (2007).

[7] Ranasinghe, R. Assessingclimate change impacts on open sandy coasts: A review. Earth-Science Reviews (2016).

[8] Van Rijn. L. C. Principles of coastal morphology. Aqua publications (1998).

[9] Bascom, W. Waves and beaches. Anchor Press/Darbleday, New York, 366 pp (1980).

[10] Eurosion. Living with Coastal Erosion in Europe: Sediment and Space for Sustainability. Part-1 Major Findings and Policy Recommendations of the EUROSION Project. Guidelines for implementing local information systems dedicated to coastal erosion management. Service contract B4-3301/2001/329175/MAR/B3 “Coastal erosion – Evaluation of the need for action”. Directorate General Environment, European Commission, 54 pp (2004).

[11] Galgano, F. A., Leatherman, S. P. & Douglas, B. C. Inlets Dominate U.S. East Coast Shoreline Change, J. Coastal Research (2004).

[12] Bird, E. C. F. Coastline changes; A Global Review. Wiley, Chichester (1985).

[13] Gorelick, N. *et al*. Google Earth Engine: Planetary-scale geospatial analysis for everyone. Remote Sensing of Environment 202 (2017).

[14] Johansen K, Phinn S, Taylor M (2015). Mapping woody vegetation clearing in Queensland, Australia from Landsat imagery using the Google Earth Engine. Remote Sensing Applications: Society and Environment.

[15] García-Rubio G, Huntley D, Russell P (2015). Evaluating shoreline identification using optical satellite images. Marine Geology.

[16] Bayram B, Acar U, Seker D, Ari A (2008). A Novel Algorithm for Coastline Fitting through a Case Study over the Bosphorus. Journal of Coastal Research.

[17] Kuleli T, Guneroglu A, Karsli F, Dihkan M (2011). Automatic detection of shoreline change on coastal Ramsar wetlands of Turkey. Ocean Engineering.

[18] Pardo-Pascual JE, Almonacid-Caballer J, Ruiz LA (2012). & Palomar-Vazquez. Automatic extraction of shorelines from Landsat TM and ETM+multi-temporal images with subpixel precis. ion. Remote Sensing of Environment.

[19] Liu Q, Trinder J, Turner IL (2017). Automatic super-resolution shoreline change monitoring using Landsat archival data: a case study at Narrabeen–Collaroy beach, Australia. J. Appl. Remote Sens..

[20] Hagenaars, G., de Vries, S., Luijendijk, A. P., de Boer, W. P. & Reniers, A. J. H. M. On the accuracy of automated shoreline detection derived from satellite imagery: A case study of the Sand Motor mega-scale nourishment. Coastal Engineering (2017).

[21] Finkl CW (2004). Coastal classification: systematic approaches to consider in the development of a comprehensive scheme. J. Coast.Res..

[22] French, J. *et al*. Conceptualising and mapping coupled estuary, coast and inner shelf sediment systems. *Geomorphology*, 10.1016/j.geomorph.2015.10.006 (2016).

[23] Inman DL, Nordstrom CE (1971). On the Tectonic and Morphologic Classification of Coasts. The Journal of Geology.

[24] Davies JL (1964). A morphogenic approach to world shorelines. Zeitschrift für Geomorphologie.

[25] Hayes, M. O. Barrier island morphology as a function of wave and tide regime. In: Barrier islands from the Gulf of St. Lawrence to the Gulf of Mexico: Academic Press, New York, NY (1979).

[26] Davies RA, Hayes MO (1984). What is a wave dominated coast?. Marine geology.

[27] Boyd R, Dalrymple RW, Zaitlin BA (1992). Classification of clastic coastal depositional environments. Sedimentary Geology.

[28] Shepard FP (1976). Coastal classification and changing coastlines. Geoscience and Man.

[29] Friedman GM (1961). Distinction between dune, beach and river sands from their textural characteristics. Journal of SedimentaryPetrology.

[30] Giri C (2011). Status and distribution of mangrove forests of the world using earth observation satellite data. Global Ecol. Biogeogr..

[31] Hayes MO (1967). Relationship between coastal climate and bottom sediment type on the inner continental shelf. Jour. Mar. Geol..

[32] Bird, E. C. F. & Schwartz, M. L. The world’s coastline. Van Nostrand Reinhold, New York (1985).

[33] Stive MJ (2002). Variability of shore and shoreline evolution. Coastal engineering.

[34] Moore LJ (2000). Shoreline mapping techniques. J. Coastal Res..

[35] Leatherman SP (1983). Shoreline Mapping: A Comparison Techniques. Shore and Beach.

[36] Esteves LS, Finkl CW (1998). The problem of critically eroded areas (CEA): An evaluation of Florida beaches. Journal of Coastal Research, SI.

[37] UNEP-WCMC & IUCN. Protected Planet Report 2016. UNEP-WCMC and IUCN: Cambridge UK and Gland, Switzerland (2016).

[38] Bird, E. C. F. Coastal geomorphology: an introduction, 2nd edn. Wiley, Chichester (2008).

[39] Anthony EJ (2015). Linking rapid erosion of the Mekong River delta to human activities. Sci. Rep..

[40] Elmoustapha. A. O., Levoy, F., Monfort, O. & Koutitonsky, V. G. A Numerical Forecast of Shoreline Evolution after Harbour Construction in Nouakchott, Mauritania. Journal of Coastal Research: Volume 23, Issue 6 (2007).

[41] Dyson A, Victory S, Connor T (2001). Sand bypassing the Tweed River Entrance: an overview. roc. Coasts and Ports Conference.

[42] Pranzini, E. C Erosion and Protection in Europe edited by Enzo Pranzini, Allan Thomas Williams (2013).

[43] Short, A. D. Handbook of Beach and Shoreface Morphodynamics. Chichester: John Wiley & Sons (1999).

[44] Heinz Center. Evaluation of erosion hazards, 203 pp., The H.John Heinz III Center for Science, Economics and the Environment,Washington, D. C. (2000).

[45] OpenStreetMap contributors. Planet dump retrieved from, https://planet.osm.org (2015).

[46] World Factbook: Coastline, United States Central Intelligence Agency. https://www.cia.gov/library/publications/the-world-factbook/fields/2060.html (2016).

[47] Burke, L. *et al*. Pilot analysis of global ecosystems: Coastal Ecosystems. *World Resources Institut*e (2001).

[48] Donchyts G (2016). Earth’s surface water change over the past 30 year. s. Nature Clim. Change.

[49] Hansen MC (2013). High-Resolution Global Maps of 21st-Century Forest Cover Change. Science.

[50] Otsu, N. A Threshold Selection Method from Gray-Level Histograms. I*EEE Transactions on Systems, Man, and Cybernetics* Vol. SMC-9, No. 1, 10.1109/TSMC.1979.4310076 (1979).

[51] Pekel, J. F., Cottam, A., Gorelick, N. & Belward, A. S. High-resolution mapping of global surface water and its long-term changes. *Nature Letter*, 10.1038/nature20584 (2016). 10.1038/nature20584 27926733

[52] Hapke, C. J. & Henderson, R. E. Quantification of Shoreline Change Along Hatteras Island, North Carolina-Oregon Inlet to Cape Hatteras, 1978–2002, and Associated Vector Shoreline Data. USGS report (2015).

[53] Crowell, M., Leatherman, S. P. & Buckley, M. K. Shoreline Change Rate Analysis: Long Term Versus Short Term Data, Shoreand Beach, pp. 13–20 (1993).

[54] Hardisty, J. Beach and nearshore sediment transport. In: Pye, K. Sediment transport and depositional processes. Blackwell, London, UK. pp. 216–255 (1994).

[55] Brown, A. C. Biology of sandy beaches. In: Encyclopedia of Ocean Sciences, Volume 5, ed. Steele, J. H., Thorpe, S. A. & Turekian, K. K., pp. 2496–2504. London, UK: Academic Press (2001).

[56] Durgappa, R. Coastal protection works. Proceedings, Seventh International Conference of Coastal and Port Engineering in Developing Countries, COPEDEC VII, Dubai (2008).

[57] Woodroffe, C. D. *et al*. Approaches to risk assessment on Australian coasts: A model framework for assessing risk and adaptation to climate change on Australian coasts. NCCAR (2012).

[58] Hunter, J. D. Matplotlib: A 2D graphics environment. Computing in Science & Engineering **9**(3), (2007).

