# Author Correction: The State of the World’s Beaches

**DOI:** 10.1038/s41598-018-28915-8

**Published:** 2018-07-24

**Authors:** Arjen Luijendijk, Gerben Hagenaars, Roshanka Ranasinghe, Fedor Baart, Gennadii Donchyts, Stefan Aarninkhof

**Affiliations:** 1 Faculty of Civil Engineering and Geosciences, Delft University of Technology, Delft, The Netherlands; 2 Deltares, Delft, The Netherlands; 3 IHE Delft, Institute for Water Education, Department of Water Science and Engineering, Delft, The Netherlands; 4 Water Engineering and Management, Faculty of Engineering Technology, University of Twente, Enschede, The Netherlands

Correction to: *Scientific Reports* 10.1038/s41598-018-24630-6, published online 27 April 2018

The Acknowledgements section in this Article is incomplete.

“This work is funded by NatureCoast, a project of technology foundation STW (applied science division of NWO) and the Deltares Strategic Research Programme ‘Coastal and Offshore Engineering’. Roshanka Ranasinghe is supported by the AXA Research Fund. We would like to thank Peter Ruggiero (Oregon State University), Cheryl Hapke (USGS) and Mitch Harley (UNSW Water Research Laboratory) for sharing the long-term *in-situ* shoreline measurements allowing for a multi-site validation. The Google Earth Engine team is acknowledged for enabling this global assessment through the GEE platform and their assistance during the analysis. We would also like to acknowledge the map data copyrighted OpenStreetMap contributors for using the map data available from https://www.openstreetmap.org.”

should read:

“This work is funded by NatureCoast, a project of technology foundation STW (applied science division of NWO) and the Deltares Strategic Research Programme ‘Coastal and Offshore Engineering’. Roshanka Ranasinghe is supported by the AXA Research Fund. We would like to thank Peter Ruggiero (Oregon State University), Cheryl Hapke (USGS) and Mitch Harley (UNSW Water Research Laboratory) for sharing the long-term *in-situ* shoreline measurements allowing for a multi-site validation. The Google Earth Engine team is acknowledged for enabling this global assessment through the GEE platform and their assistance during the analysis. We would also like to acknowledge the map data copyrighted OpenStreetMap contributors for using the map data available from https://www.openstreetmap.org. We especially would like to acknowledge Josh Friedman for his contributions during his time at Deltares, particularly in relation to automated sandy beach detection, in the initial stages of this global assessment.”

