# Supplementary material for: The State of the World’s Beaches
Source: Sci Rep. 2018 Apr 27;8:6641. doi: 10.1038/s41598-018-24630-6 (PMC5923213; doi:10.1038/s41598-018-24630-6)
Supplement: Supplementary file 1 — Supplementary material [file 41598_2018_24630_MOESM1_ESM.pdf]

Supplementary Material

The State of the World's Beaches

Arjen Luijendijk[a,b], Gerben Hagenaars[b], Roshanka Ranasinghe[c,d,b],
Fedor Baart[b], Gennadii Donchyts[a,b], Stefan Aarninkhof[a]

[a] *Faculty of Civil Engineering and Geosciences,*
Delft University of Technology, Delft, The Netherlands
[b] *Deltares, Delft, The Netherlands*
[c] *IHE Delft, Institute for Water Education, Department of Water Science and Engineering,*
Delft, The Netherlands
[d] *Water Engineering and Management, Faculty of Engineering Technology,*
University of Twente, Enschede, The Netherlands
Correspondence and requests for materials should be addressed to A.L. (a.p.luijendijk@tudelft.nl)

The supplementary material discusses the following three items:

S1. Validation of sand detection

S2. Validation of shoreline detection

S3. Quantification of local scale beach erosion / accretion

S1. Validation of sand detection

To validate the sand detection method we followed the guidelines by Richards [1] who recommends the use of 50 validation locations per class. These locations were selected independently from the training dataset and were randomly spread over the world. Figures S.1-6 show a subset of 39 sites at which validation was done via visual inspection. The yellow-coloured areas on each satellite image are the 'sandy' pixels that the supervised classification method identified as 'sandy' areas.

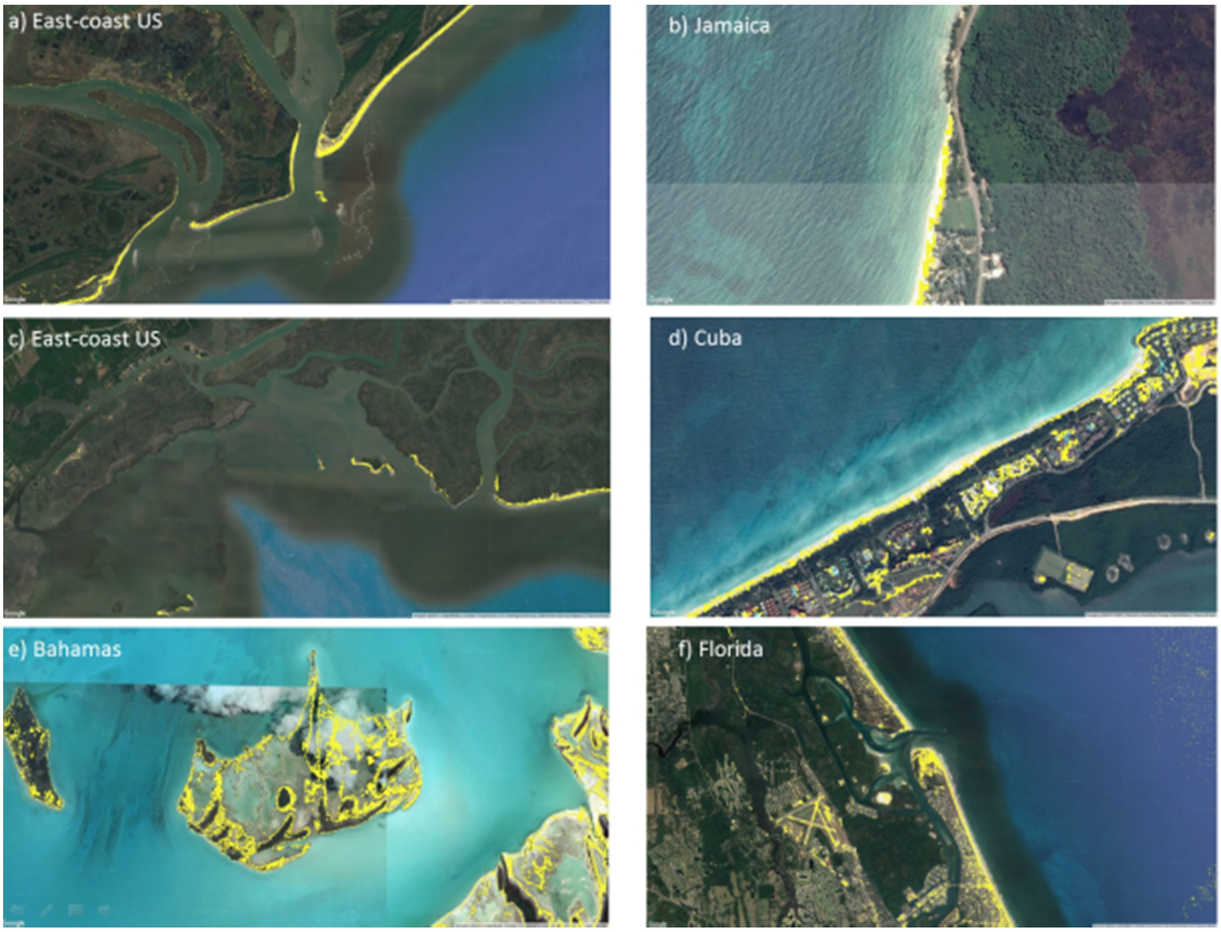

Figure S.1. Satellite images for six selected sites in North-America and Caribbean: areas detected as 'sandy' are highlighted in yellow. Maps are created with QGIS version 2.18.3 (Open Source Geospatial Foundation Project, http://qgis.osgeo.org) using satellite images provided by Google Maps. Map data: Google and DigitalGlobe.

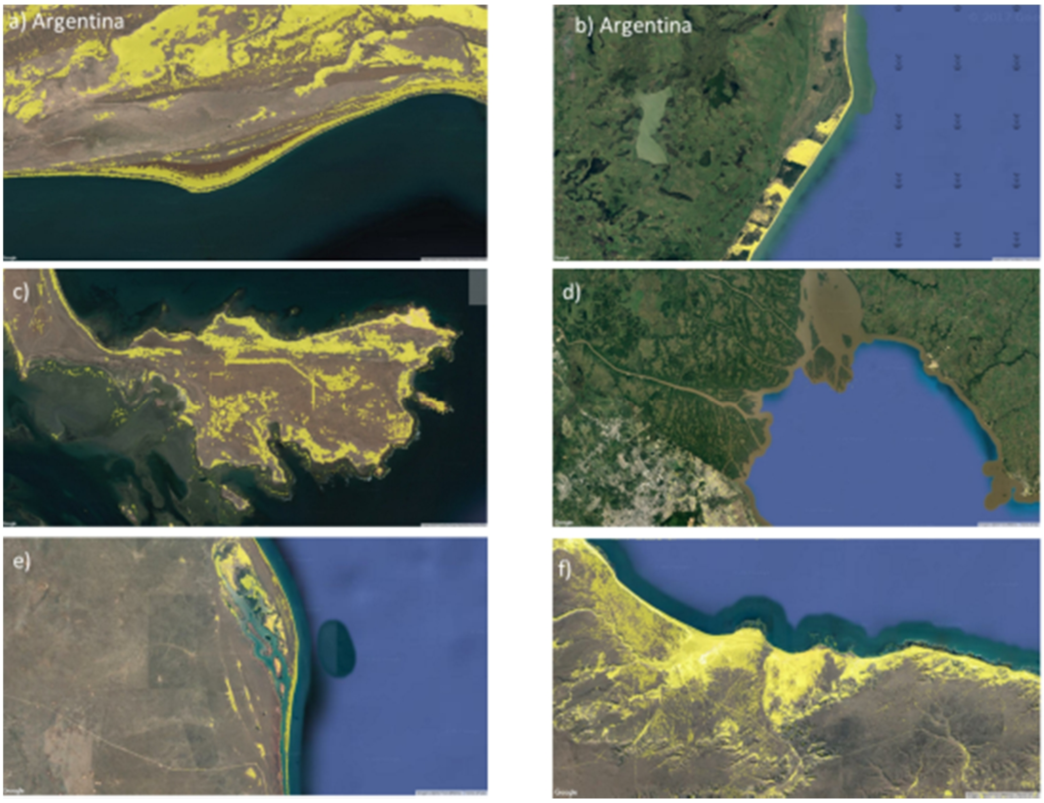

Figure S.2. Satellite images for six selected sites in South-America: areas detected as 'sandy' are highlighted in yellow. Maps are created with QGIS version 2.18.3 (Open Source Geospatial Foundation Project, http://qgis.osgeo.org) using satellite images provided by Google Maps. Map data: Google and DigitalGlobe

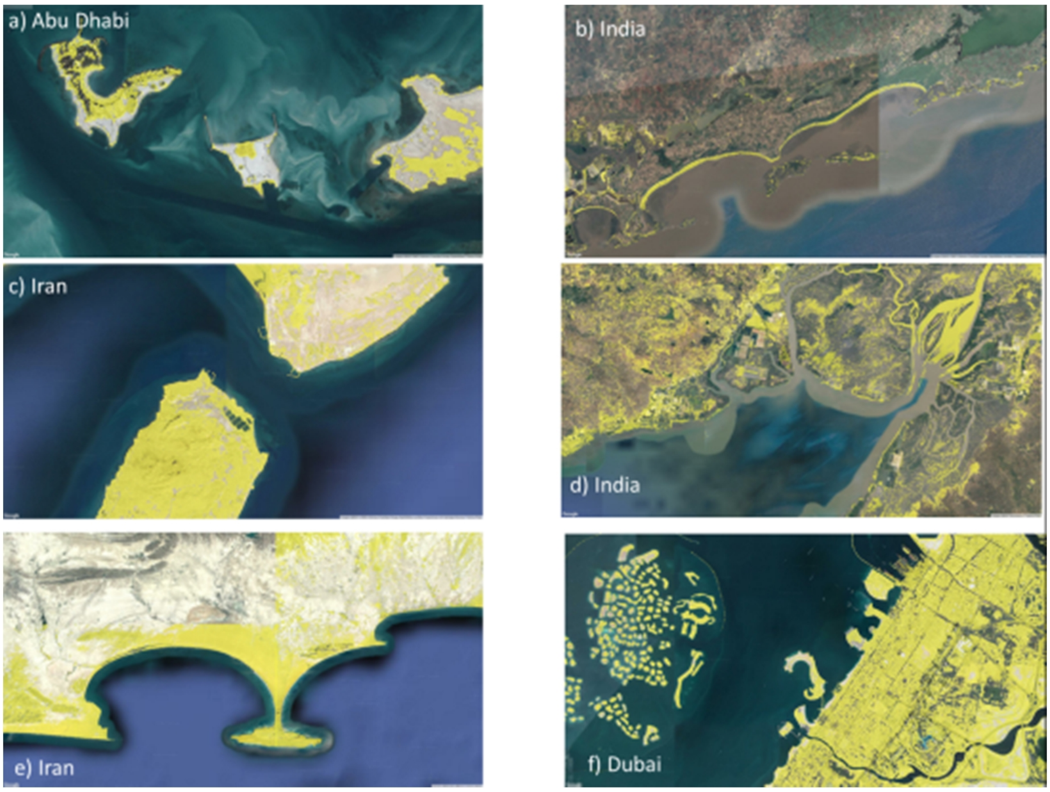

Figure S.3. Satellite images for six selected sites in the Middle East: areas detected as 'sandy' are highlighted in yellow. Maps are created with QGIS version 2.18.3 (Open Source Geospatial Foundation Project, http://qgis.osgeo.org) using satellite images provided by Google Maps. Map data: Google and DigitalGlobe

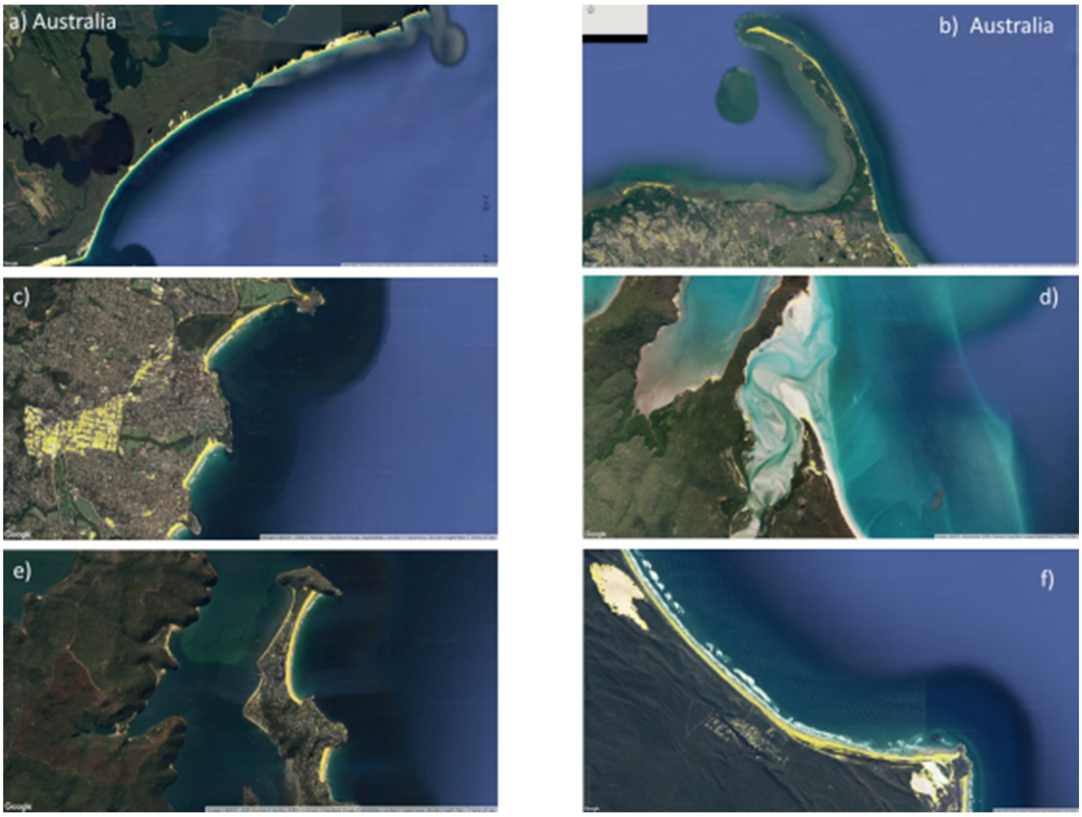

Figure S.4. Satellite images for six selected sites in Australia: areas detected as 'sandy' are highlighted in yellow. Maps are created with QGIS version 2.18.3 (Open Source Geospatial Foundation Project, http://qgis.osgeo.org) using satellite images provided by Google Maps. Map data: Google and DigitalGlobe.

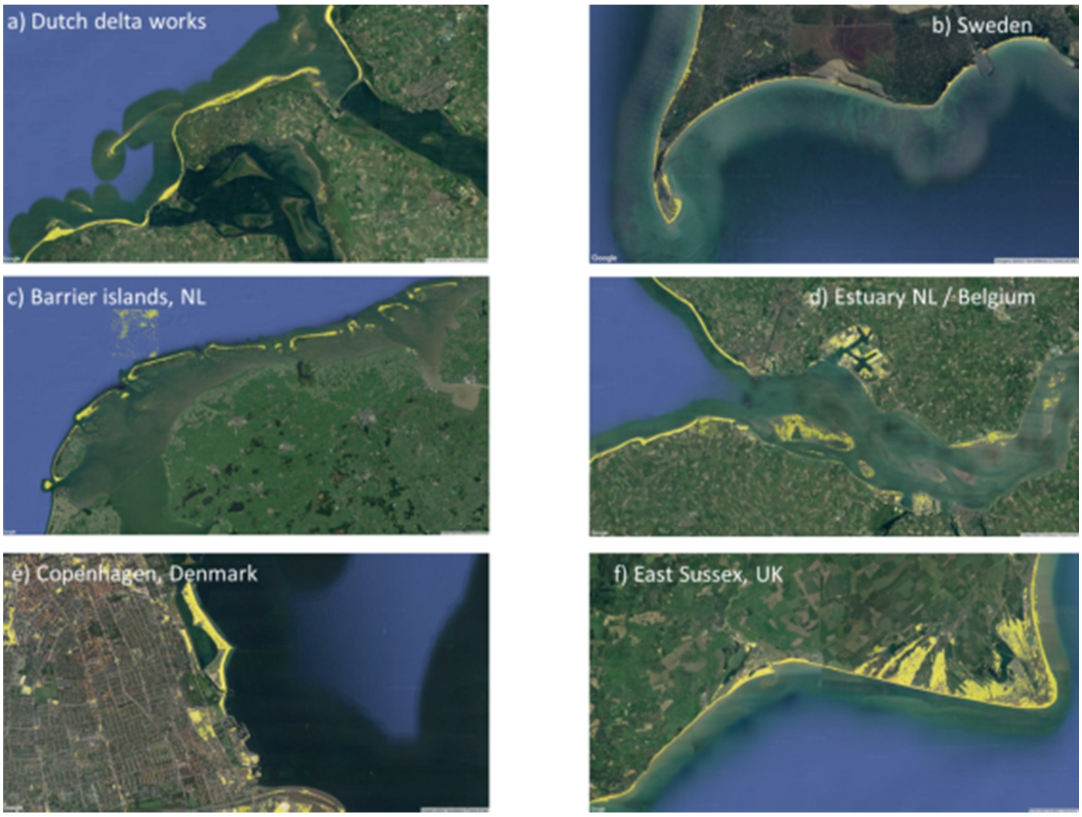

Figure S.5. Satellite images for six selected sites in Europe: areas detected as 'sandy' are highlighted in yellow. Maps are created with QGIS version 2.18.3 (Open Source Geospatial Foundation Project, http://qgis.osgeo.org) using satellite images provided by Google Maps. Map data: Google and DigitalGlobe.

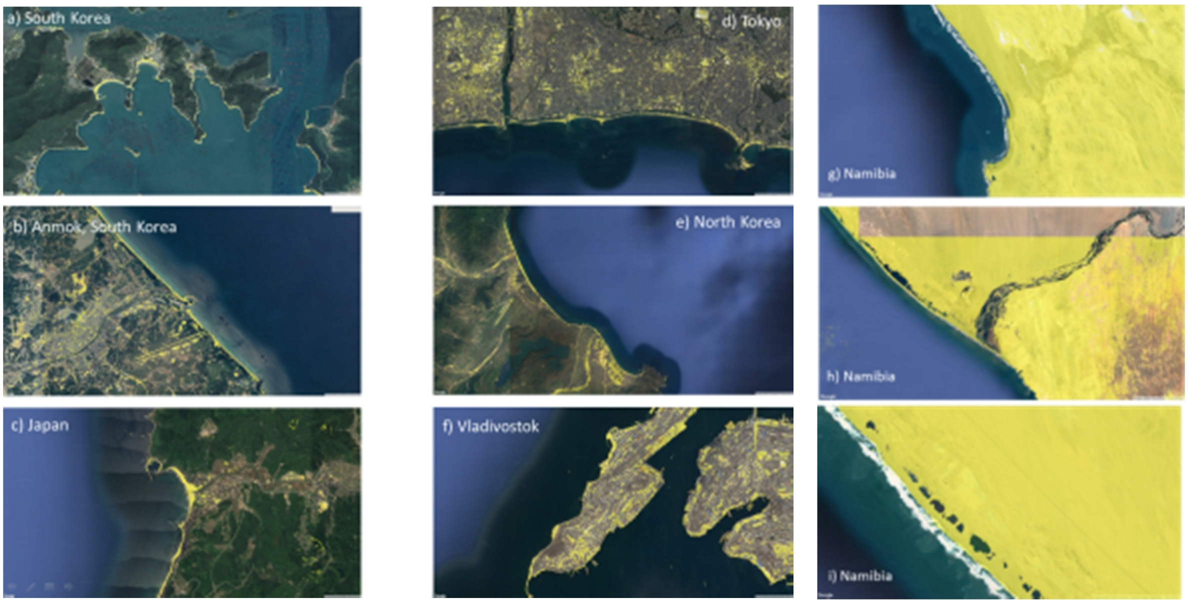

Figure S.6. Satellite images for nine selected sites in South-East Asia and Africa (Namibia): areas detected as 'sandy' are highlighted in yellow. Maps are created with QGIS version 2.18.3 (Open Source Geospatial Foundation Project, http://qgis.osgeo.org) using satellite images provided by Google Maps. Map data: Google and DigitalGlobe.

For the 'sand' class, visual inspection of the 50 validation locations resulted in 96 % accuracy. Only two sites were only partly identified as sand, although visually recognized as a fully sand beach: the white sandy Whitehaven Beach in Australia (see Figure S.4d) and part of the sandy Ras Ghurab Island in Abu Dhabi (see Figure S.3a).

S2. Validation of shoreline detection

Four sites are used to validate and evaluate the shoreline detected method covering different tidal ranges and analysis periods (see Table S.1). For the Narrabeen (Australia) and Sand Engine (The Netherlands) sites, shoreline change analysis is conducted for five and 50 profiles, respectively. For Long Beach and Hatteras Island (both in the USA) we have also analysed the alongshore variability of the shoreline change trend rates covering periods of 18 and 13 years, respectively. In total, more than 3,600 surveyed shoreline positions are considered in the validation and positional accuracy quantification.

The positional accuracy at the four sites is quantified by directly comparing surveyed shoreline positions with the concurrent position of the satellite derived shorelines (SDS). For each site, the local coordinate system of survey transects were adopted. Figure S.7 shows the resulting correlation between surveyed shorelines and SDS positions. The position of the SDS correlates well with surveyed shorelines as indicated by correlation coefficients ranging from 0.85 to 0.99 at the four sites (see Table S.1). The average of the offset values of three of four sites is 2.3 m and associated RMSE values range from half a pixel (0.5) to one pixel (1.1), which are comparable with findings of recent studies at micro tidal beaches[2,3]. Only in case of Long Beach, despite a high correlation (R2 of 0.98), a relatively large seaward mean offset was found (-35.2 m). This is attributed to the persistent presence of wave-induced foam due to breaking of swell waves along this highly energetic coastline as discussed in Section 3.3 in the manuscript. Due to its persistency, the wave-induced foam effect on detected shorelines is however likely to be limited where long-term shoreline change rates at such sites are concerned. The relatively large RMSE value (50.5 m) is most probably related to the large tidal range; yet this needs to be further verified with additional in-situ surveyed shorelines. The high correlation (R2 of 0.98) found between surveyed shoreline and SDS positions at Long Beach, demonstrates that the shoreline data is still relevant to long-term shoreline change analysis.

Table S.1. Characteristics of validation data sets and error statistics

Validation site	# of shoreline positions, # of transects, and spatial coverage	Analysis period (yrs)	Tidal range (m)	Mean offset (m)	RMSE (m)	R^2
1. Hatteras Island, USA	1234 shoreline positions<br>200 transects over ~60 km	13<br>(1989-2002)	1.7	-2.0	17.4	0.85
2. Narrabeen, Australia	1043 shoreline positions<br>5 transects over 3 km	29<br>(1986-2016)	1.9	1.1	13.7	0.88
3. Sand Engine, The Netherlands	485 shoreline positions<br>55 transects over ~10 km	4<br>(2011-2016)	2.0	-4.0	33.4	0.99
4. Long Beach, WA, USA	918 shoreline positions<br>17 transects over ~44 km	18<br>(1997-2015)	3.6	-35.2	50.5*	0.98

* *Corrected for displacement by subtracting the mean offset (due to persistent wave-induced foam) from all shoreline positions*

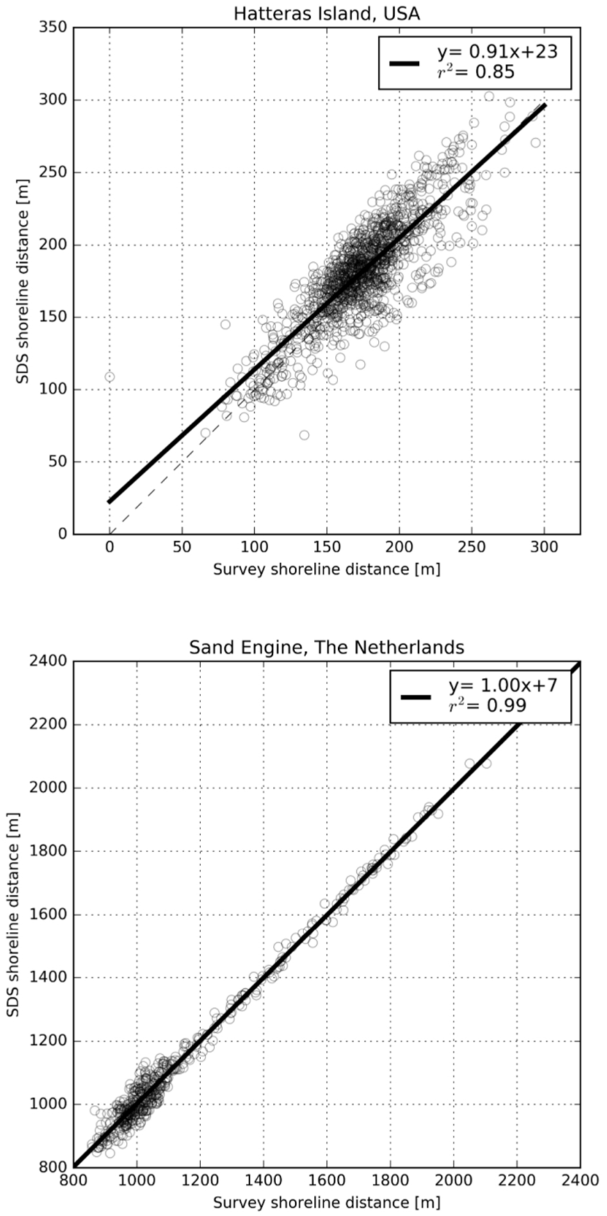

Figure S.7. Correlation between the surveyed shorelines and the SDS shorelines for the four validation sites. The linear regression fit is plotted in black, while the 1:1 line is plotted as a dashed grey line. Figure is created with Python 2.7.12 (https://www.python.org) using Matplotlib[4].

Below we present and describe time series of shoreline positions for three validation sites (Hatteras Island is already discussed in the manuscript).

Validation case 2: Narrabeen, Australia

Beach profiles have been measured at approximately monthly intervals from 1976 to date at Narrabeen-Collaroy beach, Sydney, Australia[5]. This is one of just a handful of sites worldwide where ongoing and uninterrupted beach monitoring now spans multiple decades. Monthly cross-shore beach profile surveys at five locations along the beach are here used as ground truth data to assess the accuracy of the shoreline detection method.

For the period 1984 till 2016 we collated 397 cloud-free satellite images and determined the shoreline position at the PF1 profile. The variation in shoreline positions over the three decades is approx. 90 m; i.e. equivalent to three pixels. Yet, the SDS data shows remarkable resemblance with the in-situ measured variation in shoreline positions (see Figure S.8), which is in line with the findings of Liu[6].

The linear regression trend calculated from the SDS is identical to the observed shoreline change rates. This validation case highlights the capabilities in detecting shoreline changes at subpixel level.

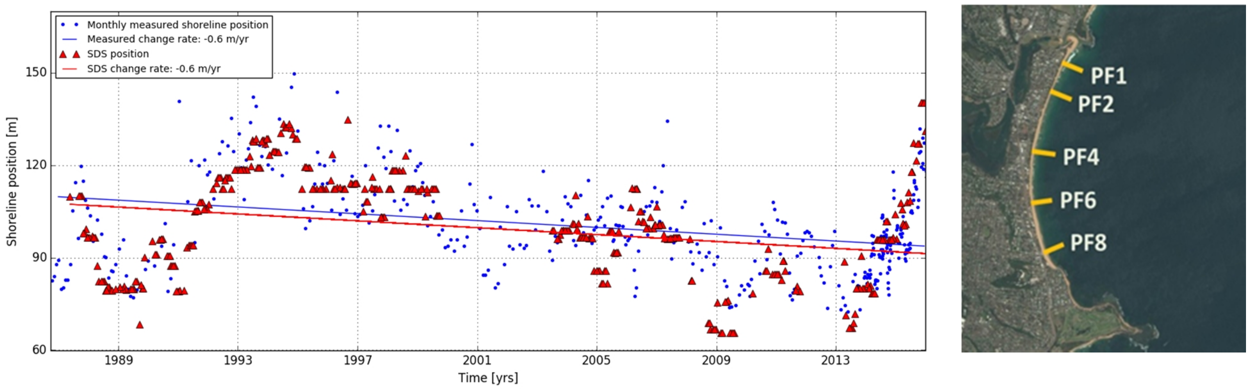

Figure S.8. In-situ measured and satellite-derived shoreline positions between 1987 and 2016 for profile PF1 (northern profile) at Narrabeen, Australia. Figure is created with Python 2.7.12 (https://www.python.org) using Matplotlib[4]. The map in the figure is made using © Mapbox and © OpenStreetMap, available under the Open Database License (https://www.openstreetmap.org/copyright).

Validation case 3: Large-scale beach nourishment (Sand Engine), The Netherlands

To assess the accuracy of the satellite derived shoreline from the satellite images the SDS should ideally be compared with a shoreline derived from in-situ measurements taken on the same day. This minimizes the effect of morphological changes on the coastline comparison. High-frequency bathymetric surveys available from a large-scale beach nourishment near The Hague, The Netherlands, enabled such a direct comparison of shorelines derived from in-situ measurements and a high-resolution SPOT satellite image on the same clear sky day (in July 2015). After tidal correction, the comparison revealed a highly accurate SDS position with a spatial mean offset of 1.0 m [2].

Monthly surveys are available after completion of the large-scale nourishment in 2011. Comparisons have been made for a series of transects of which two are presented below (see Figure S.9). The behaviour of the shoreline changes is represented well by the SDS data, making it an attractive alternative for monitoring the shoreline evolution of such sandy interventions.

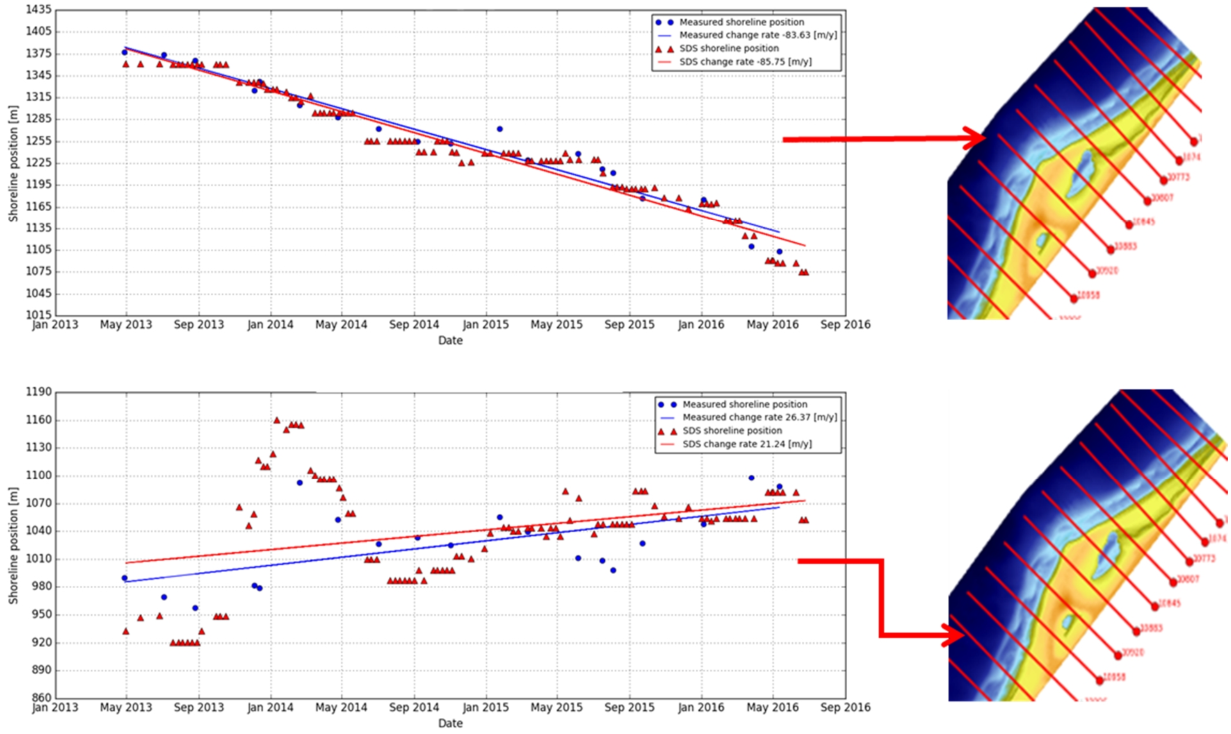

Figure S.9. In-situ measured and satellite-derived shoreline positions between 2013 and 2017 for two transects at the Sand Engine, The Netherlands. Figure is created with Python 2.7.12 (https://www.python.org) using Matplotlib[4].

Validation case 4: Long Beach, WA, USA

The Long Beach sub-cell stretches 44 km between the north jetty of the Columbia River and Leadbetter Point at the tip of Long Beach Peninsula (see Figure S.10). The initial shoreline response due to construction of the Columbia River North Jetty (1885–1913) was rapid and was confined to the development of a pocket beach between the jetty and North Head. Over the long term (1870s – 2002), the average shoreline change rate along Long Beach was 2.6 m/yr, with rates ranging from -12.1 to 10.3 m/yr [7].

Seasonal profile measurements are available at various locations in the Long Beach sub-cell. These measurements are used to compare with the SDS positions between 1997 and 2016. The in-situ data indicates that the shoreline behaviour at two example locations shows significant temporal variation around a mean accretion trend. The SDS data also captures these temporal fluctuations.

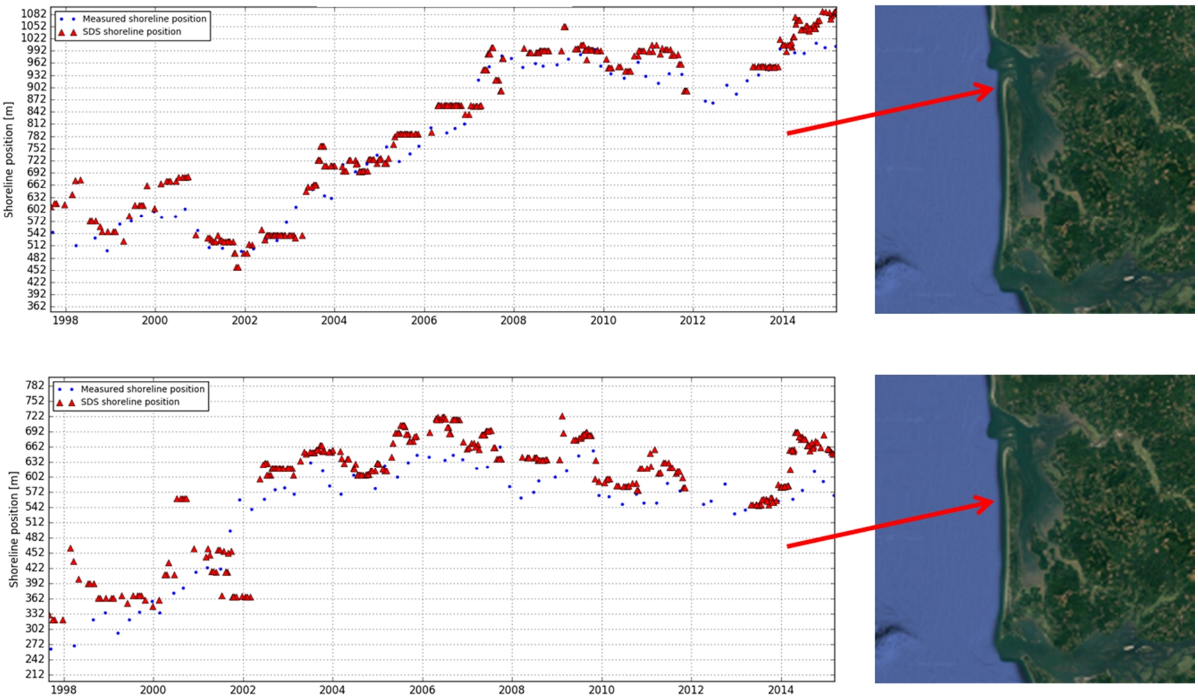

Figure S.10 In-situ measured and satellite-derived shoreline positions between 1997 and 2016 for two locations at Long Beach in Washington, USA. Figure is created with Python 2.7.12 (https://www.python.org) using Matplotlib[4]. The map in the figure is made using © Mapbox and © OpenStreetMap, available under the Open Database License (https://www.openstreetmap.org/copyright).

Linear regression is performed on the SDS data between 1997 and 2016 for each transect in the sub-cell. A similar regression approach was applied by Ruggiero[8]. The resulting trends of shoreline change rate at all transects are shown in alongshore direction in Figure S.11 for both the in-situ measurements as well as the SDS data. The alongshore distribution based on the SDS is comparable with the alongshore graph reported by Ruggiero7.

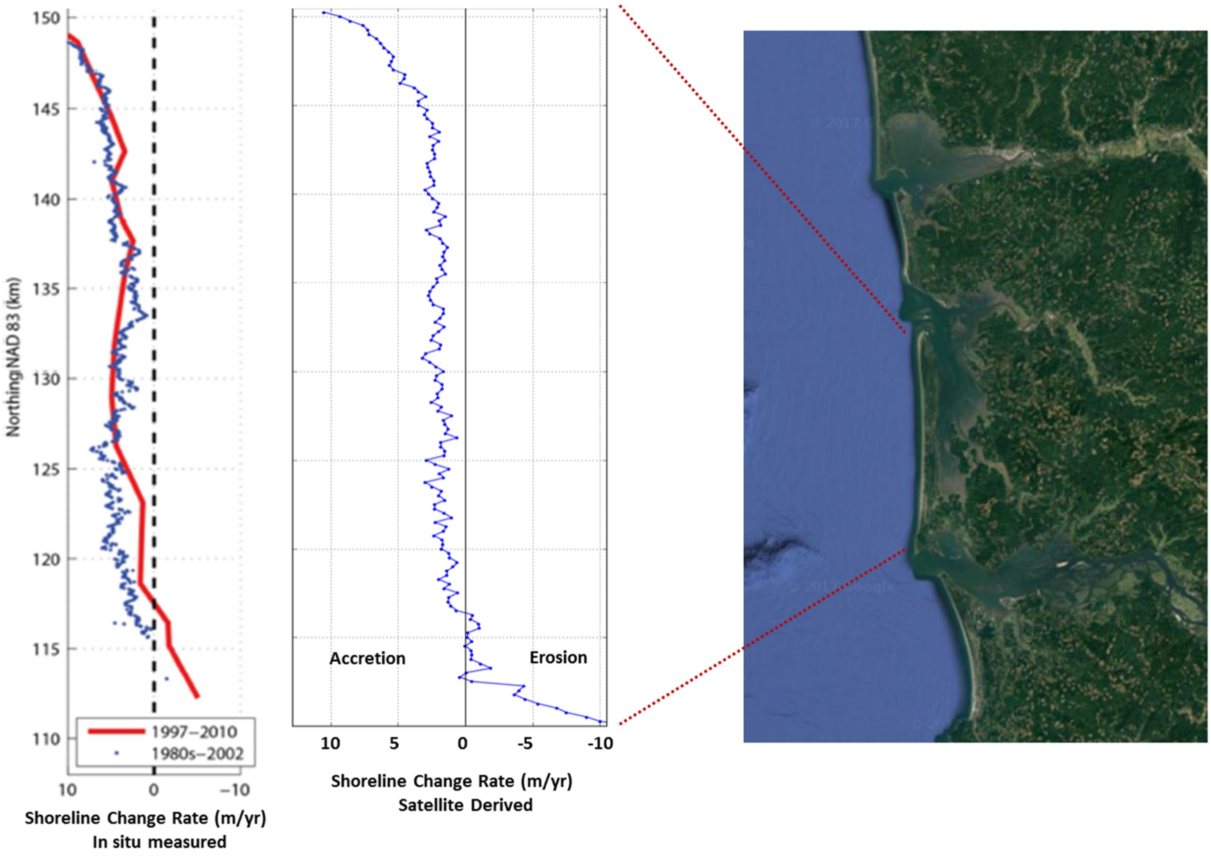

Figure S.11. Alongshore distribution of shoreline change rates along the Long Beach sub-cell in Washington, USA. Shoreline change rates are derived from in-situ measurements (panel at the left; from ref. 7) and SDS (panel in the middle) for the period 1997 - 2016. Figure is created with Python 2.7.12 (https://www.python.org) using Matplotlib[4]. The map in the figure is made using © Mapbox and © OpenStreetMap, available under the Open Database License (https://www.openstreetmap.org/copyright).

S3. Quantifying of local scale beach erosion/accretion

Below we present two highlights each of erosive/accretive behaviour from around the world, similar to Section 2.3 in the manuscript.

a) Erosion due to River damming

Since the construction of Aswan High Dam in 1964 the Nile Delta in Egypt has been seriously impacted due to the change in water and sediment flow regimes. Based on shoreline observations of 1990 and 2013, extreme erosion at the mouth of the Rosetta branch were found, with erosion rates up to ~25 m/yr [9]. Analysis of the SDS data suggests that the erosional process increased around the 2000's, with a shoreline erosion of approx. 200 m in ~12 years (Figure 12a). This example shows that SDS data can reveal fingerprints of human interventions on the coastal dynamics in time and space.

b) Erosion due to Extreme events

Hurricane Sandy made landfall at the East coast of the US in 2012, resulting in a breach of the barrier island at Fire Island, New York[10]. The storm, with offshore wave heights up to 10 m, raised the local water level by more than 4.5 m. Surveys revealed that exposed parts of the dunes that are usually not impacted by waves, experienced significant dune erosion[11]. This caused sediment deposition in the foreshore resulting in local shoreline advancement after the storm. The SDS data (Figure S.12b) shows the hurricane-induced beach accretion at this location, but also the local post-hurricane beach recovery in the years after. Interestingly, the SDS data of this transect indicate that the beach width at this location became wider after the hurricane than it had been in the preceding three decades.

c) Accretion due to sudden changes in fluvial sediment supply

The phased removal of two large dams in 2012 in the Elwha River, Washington, as part of the river-restoration project aimed at stimulating habitat restoration, resulted in sudden availability (for fluvial transport) of about 21 million m^3 of sediment that had been impounded in the reservoirs[12]. This has resulted in large volumes of fluvial sediment being deposited at the river mouth[12]. The SDS data (Figure S.12c) shows the development of an ebb tidal delta since 2014. Beach growth rates of ~15 m/yr are found in the first years after removal.

d) Accretion due to Ebb delta dynamics

Due to a closure of an estuary in the southwest of the Netherlands the relict of the corresponding ebb-tidal delta are migrating landward. The adjacent beaches are fed by the erosion of the delta front of ebb-tidal delta, which in 2005 resulted in the merger of a nearshore sand bank with the beach near Ouddorp[13]. The SDS data (Figure 12d) shows the spatio-temporal characteristics of the beach accretion which led to significant beach widening of up to 500 m in a time span of just five years.

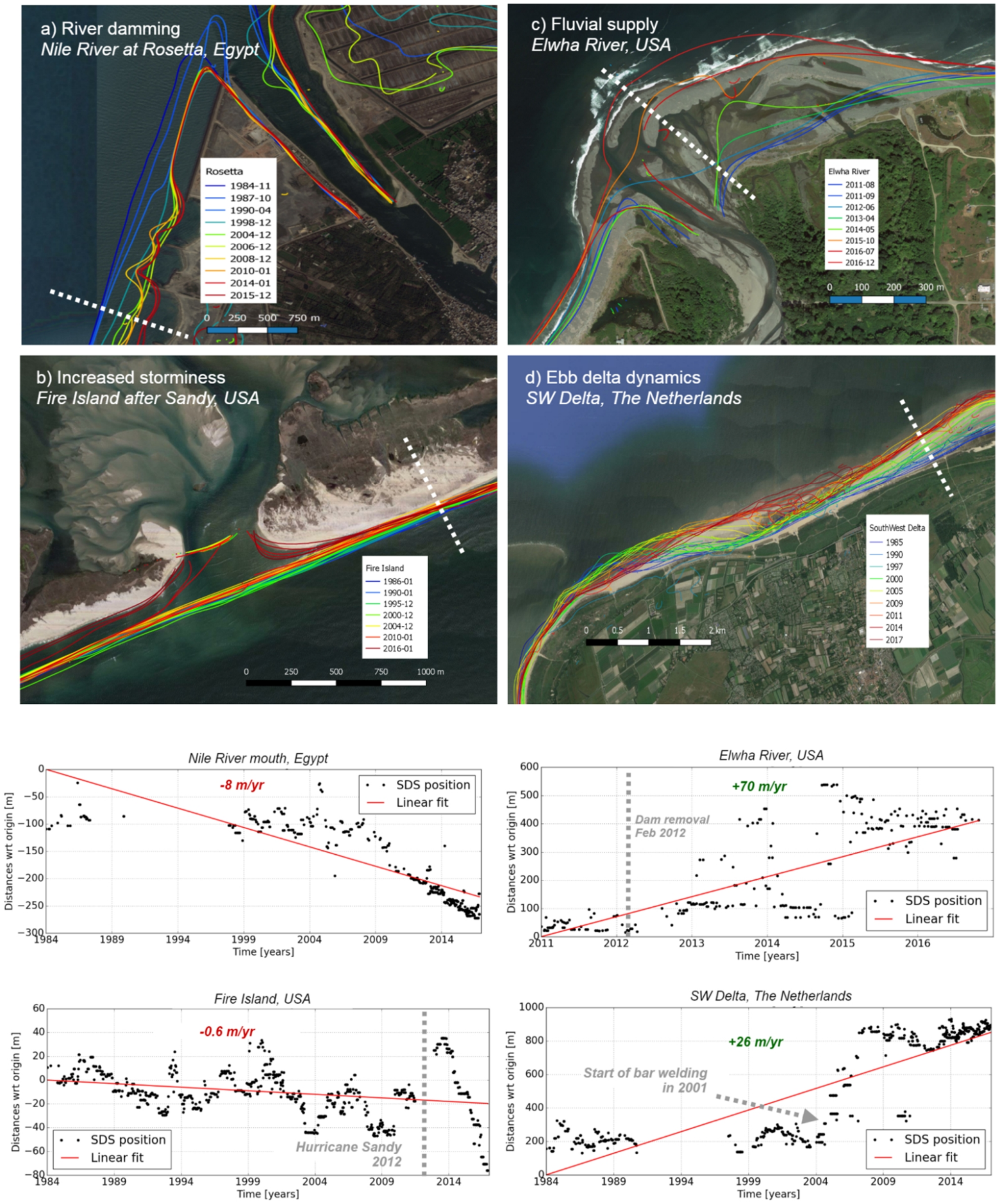

Figure S.12. Examples of the satellite derived shorelines for four selected cases of beach erosion/accretion. The left column presents two erosive cases while the right column shows two accretive cases. In each figure, the blue line indicates the oldest SDS shoreline while the red line is the most recent SDS shoreline. The graphs below indicate the shoreline positions over time at the white dashed transect for each case; the upper graphs correspond to the images on the upper row. The indicated change rates (m/yr) are obtained from fitting a line-of-best fit to the shoreline position data for each transect. Figure is created with Python 2.7.12 (https://www.python.org) using Matplotlib[4]. Maps are created with QGIS version 2.18.3 (Open Source Geospatial Foundation Project, http://qgis.osgeo.org) using satellite images provided by Google Maps. Map data: Google, CNES / Airbus, USDA Farm Service Agency, Aerodata International Surveys, Landsat / Copernicus, and DigitalGlobe.

References

[1] Richards, J.A. & Jia, X. Remote Sensing Digital Image Analysis, An Introduction. 4th edition, Springer, Germany (2005).

[2] Hagenaars, G., de Vries, S., Luijendijk, A.P., de Boer, W.P., & Reniers, A.J.H.M. On the accuracy of automated shoreline detection derived from satellite imagery: A case study of the Sand Motor mega-scale nourishment. Coastal Engineering (2017).

[3] Pardo-Pascual, J.E. et al. Assessing the Accuracy of Automatically Extracted Shorelines on Microtidal Beaches from Landsat 7, Landsat 8 and Sentinel-2 Imagery. Remote Sens. 10, 326 (2018).

[4] Hunter, J.D. Matplotlib: A 2D graphics environment. Computing in Science & Engineering 9(3), (2007).

[5] Turner, I. L. et al. A multi-decade dataset of monthly beach profile surveys and inshore wave forcing at Narrabeen, Australia. Sci. Data 3:160024 doi: 10.1038/sdata.2016.24 (2016).

[6] Liu, Q., Trinder, J., & Turner, I.L. Automatic super-resolution shoreline change monitoring using Landsat archival data: a case study at Narrabeen–Collaroy beach, Australia. J. Appl. Remote Sens. 0001;11(1):016036. doi:10.1117/1.JRS.11.016036 (2017).

[7] Ruggiero, P., Kaminsky, G.M., & Hacker, S. Morphodynamics of prograding beaches. Conference Proceeding for Coastal Dynamics 2013 (2013).

[8] Ruggiero, P., *et al*. National assessment of shoreline change: historical shoreline change along the Pacific Northwest coast. USGS Open-File Report 2012-1007. , http://dx.doi.org/10.3133/ofr20121007 (2012).

[9] Ali, E., & El-Magd, I.A. Impact of human interventions and coastal processes along the Nile Delta coast, Egypt during the past twenty-five years. The Egyptian Journal of Aquatic Research Volume 42, Issue 1 (2016).

[10] Hapke, C.J., Brenner, O.T., Hehre, R., & Reynolds, B.J. Coastal change from Hurricane Sandy and the 2012–13 winter storm season—Fire Island, New York: U.S. Geological Survey Open-File Report 2013–1231, 37 p., http://pubs.usgs.gov/of/2013/1231/ (2013).

[11] Henderson, R.H., Hapke, C.J., Brenner, O.T., & Reynolds, B.J. Hurricane Sandy beach response and recovery at Fire Island, New York: Shoreline and beach profile data, October 2012 to October 2014: U.S. Geological Survey Data Series 931, http://dx.doi.org/10.3133/ds931 (2015).

[12] Warrick, J.A. et al. Large-scale dam removal on the Elwha River, Washington, USA: source-to-sink sediment budget and synthesis. Geomorphology, 246, pp. 729-750 (2015).

[13] Elias, E.P.L., van de Spek, A.J.F., Wang, Z.B., & de Ronde, J. Morphodynamic development and sediment budget of the Dutch Wadden Sea over the last century. Netherlands Journal of Geosciences, 91(3); 293-310 (2012).
